# Long-read metagenomic exploration of extrachromosomal mobile genetic elements in the human gut

**DOI:** 10.1186/s40168-019-0737-z

**Published:** 2019-08-27

**Authors:** Yoshihiko Suzuki, Suguru Nishijima, Yoshikazu Furuta, Jun Yoshimura, Wataru Suda, Kenshiro Oshima, Masahira Hattori, Shinichi Morishita

**Affiliations:** 1 Department of Computational Biology and Medical Sciences, Graduate School of Frontier Sciences, The University of Tokyo, Chiba, 277-8568 Japan; 2 AIST-Waseda University Computational Bio Big-Data Open Innovation Laboratory, Tokyo, 169-8555 Japan; 3 Graduate School of Advanced Science and Engineering, Waseda University, Tokyo, 169-8555 Japan; 4 Division of Infection and Immunity, Research Center for Zoonosis Control, Hokkaido University, Sapporo, 001-0020 Japan; 5 Laboratory for Microbiome Sciences, RIKEN Center for Integrative Medical Sciences, Yokohama, 230-0045 Japan

## Abstract

**Background:**

Elucidating the ecological and biological identity of extrachromosomal mobile genetic elements (eMGEs), such as plasmids and bacteriophages, in the human gut remains challenging due to their high complexity and diversity.

**Results:**

Here, we show efficient identification of eMGEs as complete circular or linear contigs from PacBio long-read metagenomic data. De novo assembly of PacBio long reads from 12 faecal samples generated 82 eMGE contigs (2.5~666.7-kb), which were classified as 71 plasmids and 11 bacteriophages, including 58 novel plasmids and six bacteriophages, and complete genomes of five diverse crAssphages with terminal direct repeats. In a dataset of 413 gut metagenomes from five countries, many of the identified plasmids were highly abundant and prevalent. The ratio of gut plasmids by our plasmid data is more than twice that in the public database. Plasmids outnumbered bacterial chromosomes three to one on average in this metagenomic dataset. Host prediction suggested that Bacteroidetes-associated plasmids predominated, regardless of microbial abundance. The analysis found several plasmid-enriched functions, such as inorganic ion transport, while antibiotic resistance genes were harboured mostly in low-abundance Proteobacteria-associated plasmids.

**Conclusions:**

Overall, long-read metagenomics provided an efficient approach for unravelling the complete structure of human gut eMGEs, particularly plasmids.

**Electronic supplementary material:**

The online version of this article (10.1186/s40168-019-0737-z) contains supplementary material, which is available to authorized users.

## Background

Culture-independent metagenomics has provided a powerful approach to comprehensively explore microbial species and genes, which underlie an understanding of the ecological and biological features of the human gut microbiome [1–4]. The metagenomes of microbial communities mainly comprise bacterial chromosomes and the associated extrachromosomal mobile genetic elements (eMGEs), such as plasmids and bacteriophages (phages). These eMGEs play important roles in microbial evolution, adaptation of the community to environmental changes, and interaction with hosts by conferring a variety of accessory functions on the community [5–8]. For the analysis of plasmid communities (plasmidome), several specific procedures have been developed, including enrichment of closed circular plasmids by selective DNase treatment and CsCl-gradient ultracentrifugation from samples containing large amounts of linearized chromosomal DNAs [9, 10]. For the bacteriophage community (phageome or virome), a crucial step is the enrichment of viral particles (VLPs) from samples containing vast numbers of microbial cells. VLP preparation requires several laborious techniques, such as stepwise filtration with different pore sizes and centrifugation under adjusted gravity conditions [11–17]. However, these practices have not been well evaluated with respect to the quality and quantity of output data regarding the whole community structure.

It is also challenging to perform metagenomic sequencing of eMGE-enriched samples with short-read sequencers (Illumina and Ion Torrent) that can produce reads of only < 500 bp. For example, de novo assembly of short reads generates notably short linear contigs [3, 4], possibly due to existing homologous sequences among eMGEs and between eMGEs and chromosomes in a community. Such insufficient assembly makes it difficult to reconstruct full eMGEs as circular contigs (CCs), a structural hallmark of eMGEs excepting rare linear plasmids from metagenomic data, though there have been informatics tools that further connect the contigs to generate large bins [3, 18]. Therefore, most metagenomic studies based on short reads have analysed the whole community structure with little emphasis on separating microbial chromosomes and eMGEs [19, 20].

In contrast, long-read sequencers (Pacific Biosciences and Oxford Nanopore Technology) can produce long reads of ~ 10 kb or more. De novo assembly of long reads facilitates the generation of longer contigs and bins than those of short-read assembly by distinguishing among homologous sequences [21–26]. In addition, PacBio long-read metagenomics can also provide links between detected plasmids and their hosts using DNA methylation information [27]. However, to date, there have been no intensive long-read metagenomic studies of eMGEs [19, 20], indicating that human gut eMGEs remain to be explored. Therefore, we performed long-read metagenomics of whole faecal DNA samples to efficiently recover eMGEs as complete CCs from the assembled contigs and evaluated the diversity in human gut plasmids in this study.

## Results

### Metagenomic sequencing of human faecal samples with the PacBio SMRT system

We sequenced 13 faecal DNA samples from 12 healthy Japanese adults, including one biological duplicate (ES1-2 and ES9-1). A total of ~ 11 Gb per sample with an average subread length of 8 kb was obtained from 10 individuals (excluding two subjects with poor subread lengths) with the PacBio RS II system. We also generated short reads from six of the 12 subjects with three short-read sequencers (Illumina, 454 and Ion PGM) and obtained them from a previous publication for the other six subjects [20]. The sequencing statistics are summarised in Additional file 2: Table S1.

**Fig. 1:**
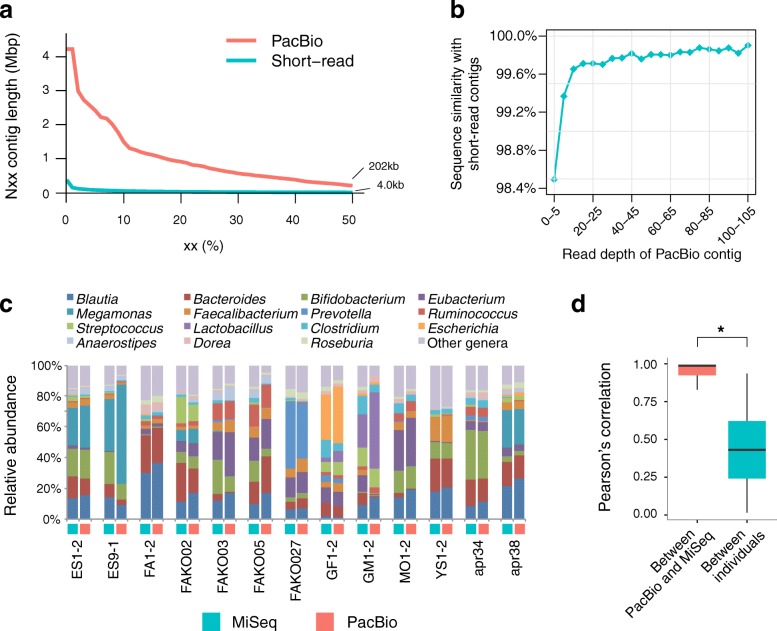
Statistics of metagenomic sequencing of 13 faecal samples with the PacBio SMRT system and short-read sequencers. **a** To show the length distribution of the contigs of long and short reads, we selected three samples (apr34, apr38, and FAKO02) that had similar sequence amounts in both PacBio long-read and short-read sequencing (see the “Results” section). The *y*-axis shows the Nxx contig length, an indicator of measuring the quality of genome assembly such that xx% of all bases in the assembled contigs of the three selected samples are found in contigs of the Nxx contig length or more, while the *x*-axis shows the value of xx, which measures coverage of bases by contigs. **b** Sequence similarity between PacBio and short-read contigs. The *y*-axis shows the sequence similarity of the PacBio contigs with the reciprocally best-matched short-read contigs, and the plots show the average value for every five units of read depth of the PacBio contigs on the *x*-axis. PacBio and short-read contigs of the 12 samples were aligned using NUCmer with a > 95% identity and a > 95% length coverage. **c** Genus-level microbial compositions estimated from the PacBio and MiSeq data of the 13 samples. Taxonomic assignment and quantification of microbial abundance from the PacBio and MiSeq data were described in the “Methods” section. **d** Pearson’s correlation coefficients (PCCs) between the microbial compositions estimated from PacBio and MiSeq data. PCCs (left) between the same samples, excluding the biological replicates (ES1-2 and ES9-1), and PCCs (right) between different samples are shown. The boxes represent the inter-quartile range (IQR), and the lines inside represent the median. The whiskers show the lowest and highest values within 1.5 times the IQR. Asterisks represent *P* < 0.01 (Wilcoxon rank-sum test)

We, therefore, conducted de novo assembly of the PacBio and short reads by using FALCON and MEGAHIT as assemblers, respectively (see the “Methods” section). We compared the two assembly outcomes from the data of three samples (apr34, apr38, and FAKO02) with similar sequence amounts in PacBio and short-read sequencing. The comparison revealed that PacBio reads boosted assembly statistics, with an N50 contig length reaching ~ 202 kb, while those of the short reads were ~ 4 kb (Fig. 1a). The results of the long-read assemblies showed that the N50 contig length ranged from 24.6 to 279.2 kb for all the samples (Additional file 2: Table S2). We then evaluated the accuracy of the PacBio contigs based on the sequence similarity between PacBio and the corresponding short-read contigs of the same samples. The results revealed that PacBio contigs with 5, 10, 20, and ≥ 40 read depths were aligned with short-read contigs with 99.4, 99.7, 99.8, and ≥ 99.9% identities, respectively (Fig. 1b). Assuming the accuracy of the aligned short-read contigs to be sufficiently high, the accuracy of PacBio contigs with read depths > 5 could be estimated to be > 99.4%, accounting for ~ 99.8% of the total contig length (Additional file 2: Table S3).

### Microbial and gene composition in PacBio metagenomic data

We compared the microbial abundance estimated from the PacBio and MiSeq reads. Taxonomic assignment of PacBio data was performed by similarity search of genes predicted in PacBio contigs for the reference genomes, followed by counting the number of PacBio reads mapped to the genes to quantify their abundance (see the “Methods” section), while that of the MiSeq data was performed by direct mapping to the reference genomes as described previously [20]. The estimated microbial abundances between the two data points in each subject were significantly similar at the genus level, with a median Pearson’s correlation coefficient of ~ 0.99, which was significantly higher than that among the 12 individuals (Fig. 1c, d).

The mean gene length in the PacBio contigs was 847 bp, longer than the 662 bp in the short-read contigs and closer to the 957 bp of mostly full-length genes in the reference genomes (Additional file 1: Figure S1a). In addition, an average of 27.6 genes was identified per PacBio contig, which was ~ 10 times more than the 2.4 per short-read contig on average (Additional file 1: Figure S1b).

### Circular contig generation from PacBio read assembly

In the assembly, we set the minimum overlap length between two subreads to 2200 bp (see the “Methods” section), though circular contigs (CCs) smaller than the cutoff (2.2 kb) cannot be identified by this method. The assembly generated a total of 82 CCs ranging from 2.8- to 666.7-kb in length (Additional file 2: Table S4). To test whether these CCs were eMGEs, we classified them as plasmids and phages using several classification assessments, such as searching phage orthologous groups (POGs, Additional file 1: Figure S2) [28], VirSorter [29], and PlasFlow [30], checking the presence or absence of known plasmid-enriched genes, such as mobilisation- and conjugation-related genes, and a similarity search of the public database. Because the POG and VirSorter assessments classified 11 CCs (30.2 to 98.9 kb in size) as phages with high consistency, we classified the remaining 71 CCs as plasmids (2.8 to 666.7 kb). A similarity search of the public plasmid/phage database revealed that 17 of the 71 plasmid CCs were highly similar to 10 known plasmids, and five of the 11 phage CCs were highly similar to a genome of a crAssphage, NC_024711.1 [31].

To further confirm the accuracy of the classifications, we blasted the CCs against the virome databases VirSorter and IMG/VR [32, 33]. The five CCs assigned to crAssphage and a putative novel phage CC (FAKO05_000032F) hit several sequences in the virome databases, consistent with the present classification. However, five plasmid-classified CCs (FA1-2_2760, FAKO05_2268, FAKO05_2271, FAKO27_6410, and FA1-2_000589F) matched sequences in the virome databases (Additional file 2: Table S4), showing disagreement with the present classification (see the “Discussion and conclusions” section).

**Fig. 2:**
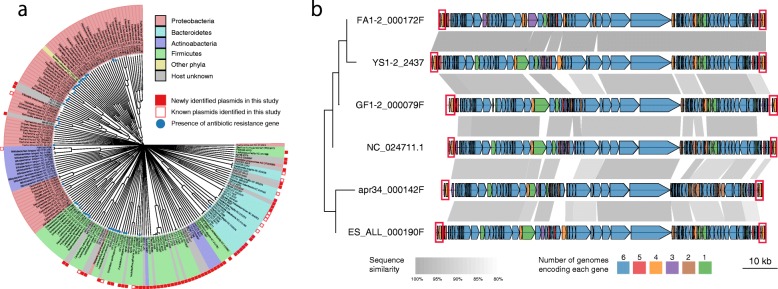
Whole-sequence comparison of 71 plasmid CCs and structure of six crAssphage linear genomes. **a** Dendrogram of 71 plasmid CCs and 114 known plasmids that were relatively abundant in the human gut (see the “Methods” section). The phyla are shown in different colours (green for Firmicutes, purple for Actinobacteria, red for Proteobacteria, blue for Bacteroidetes, yellow for other phyla and grey for unknown hosts). Red squares in the outer circle indicate the plasmid CCs newly identified in this study. Blue circles on the edges show the presence of antibiotic resistance genes. **b** Putative genes shown by pentagons in the linearized genomes of five crAssphages identified in this study and NC_024711.1 [32]. The conserved genes are connected by grey rectangles, of which shades indicate the degree of sequence similarity between them. The left dendrogram shows a clustering of the six genomes based on overall similarity. To show the degree of conservation of each putative gene in the six genomes, six different colours are used. Brown genes are unique to only one genome, while blue genes are shared in common by all genomes. The red boxes at the ends indicate TDRs in the linear genomes

We clustered the 71 plasmid CCs with 114 known plasmids relatively abundant in the human gut based on overall sequence similarity (Fig. 2a, see the “Methods” section). The results revealed that many of the 71 CCs had high sequence diversities for the known plasmids. Based on the host taxa of the known plasmids, most of the 71 CCs aggregated in Firmicutes and Bacteroidetes plasmids, and many of the novel CCs aggregated in Firmicutes plasmids, while only four novel CCs aggregated in Proteobacteria plasmids.

We also identified two highly homologous but distinct plasmid CCs in the assemblies of long reads from three subjects (apr34, FAKO03, and FAKO05). The two homologous CCs in each subject had a sequence alignment of length > 1 kb with > 99% identity between them, but in the short-read assembly, either the corresponding sequences were fragmented into multiple contigs or only one of the two CCs was generated (Additional file 1: Figure S3). These results demonstrated that homologous plasmids hard to distinguish in short-read assembly can be precisely reconstructed as independent contigs in long-read assembly. Overall, we identified 82 CCs and classified them as 71 plasmids and 11 phages, of which 58 plasmid and six phage CCs are likely to be novel eMGEs (Additional file 1: Figure S4).

We further performed the functional annotation of genes in the 71 plasmid CCs using the Clusters of Orthologous Groups (COG) database. The data revealed that ~ 47% of the genes identified were novel, and genes assigned to COG category X, “Microbiome”, were most enriched in the functionally annotated genes, as expected (Additional file 2: Table S5).

### Structure of contigs assigned to the crAssphage genome

Mapping of PacBio and short reads to the five crAssphage CCs suggested that these CCs had a linear genome with terminal direct repeats (TDRs) of length ~ 2 kb. This was supported by several lines of evidence, e.g., ~ twofold higher coverage of both PacBio and short reads mapped to the TDR region than other regions in the circular genome, absence of PacBio reads spanning the TDRs, and higher frequency of both PacBio and short reads starting from both ends of the TDRs than reads from other positions (Additional file 1: Figure S5). Both TDRs in each genome were almost identical, while the sequence similarity and length slightly varied among TDRs in the five crAssphages (Additional file 2: Table S6 and Additional file 1: Figure S6). The linear genomes of six crAssphages, including NC_024711.1, encoded 89 to 91 putative genes, of which 61 were highly conserved with ≥ 80% amino acid identity among them; the number of genes unique to each genome ranged from 0 to 16 with an average of 6.3 per genome, and other conserved genes numbered between two and five (Fig. 2b). Additionally, the genomes exhibited a clear transition in GC skew of the coding strand at approximately 30 kb away from the right TDR (Additional file 1: Figure S7). Similarly, two phage CCs (FAKO05_000032F and FAKO27_000271F) were found to have linear genomes by mapping the reads to the CCs (Additional file 1: Figure S8). Our data indicated that linear phage genomes with TDRs were erroneously assembled as CCs. The TDRs are the source of this mis-assembly, which could be corrected by mapping the reads to CCs as described previously [34].

### Reconstruction of microbial chromosomes from PacBio contigs

The assembly of PacBio reads also yielded seven large CCs from 2 to 3 Mb in length, which were considered to be bacterial chromosomes. We additionally reconstructed 94 high-quality (HQ) chromosome bins (completeness > 90%, contamination < 5%) with putative genome sizes ranging from 1.88 to 6.83 Mb, in which multiple rRNA genes were consistently allocated (Additional file 2: Table S7). Of these chromosome bins, 17 might be phylogenetically novel, because their identities with known genomes were lower than the threshold (96.5%) [35]. Phylogenetic tree analysis indicated that 69 bins, including the 17 novel bins, were taxonomically classified as Firmicutes, 18 as Bacteroidetes, 13 as Actinobacteria, and one as Proteobacteria (Additional file 1: Figure S9).

### Host prediction of eMGEs

Host prediction of the 82 eMGEs was performed by several methods: sequence similarity search for publicly available draft genomes [36], co-occurrence profile based on abundance (CO) [31], methylation motif (MM) similarity [27], and CRISPR spacer similarity to only the phage’s host [37, 38].

A similarity search of the 71 plasmid CCs for the draft genomes showed that 36 CCs hit the draft genomes of various strains, which were taxonomically well-matched with those assigned by the similarity search for known plasmids (Additional file 2: Tables S8 and S9). In the host prediction by CO analysis, we used the IGCJ dataset composed of 413 faecal metagenomic data from Japan (JP), the US (US), Spain (ES), Denmark (DK), and China (CN) (see the “Methods” section) [19, 20]. We identified nine CCs that had Spearman’s correlation coefficients [31] of > 0.7 for variance in abundance with several genomes/hosts across the samples (Additional file 2: Table S9). The MM similarity search using the present JP PacBio dataset found 19 plasmid CCs that shared 26 different MMs with 14 HQ chromosome bins (Additional file 1: Figure S10 and Additional file 2: Table S9).

As shown in Fig. 2a, many of the plasmids, including the host-predicted plasmid CCs, tended to be grouped by host taxa, except for the five Actinobacteria-predicted novel CCs that segregated from the known Actinobacteria plasmids.

We further constructed a host-plasmid network from the host-predicted plasmid CCs and found many shared plasmids between various *Bacteroides* species and several *Parabacteroides* and *Prevotella* species, forming a large network distinct from others in the human gut microbiomes of the 12 subjects (Additional file 1: Figure S11).

In the host prediction of phages, because no host candidate was identified in the CO analysis and the similarity search, we used three different datasets (JP PacBio, IGCJ, and the public genome database) for CRISPR spacer similarity search and the JP PacBio dataset for the MM similarity search. Four phage contigs (FAKO27_000271F, YS1-2_2434, FAKO27_000238F, and apr34_1784) had nearly perfect matches with CRISPR spacers in several genomes of the three datasets (Additional file 2: Table S10) and concurrently shared 13 MMs with four genomes in the JP PacBio dataset (Additional file 1: Figure S10). The hosts of the four phages as predicted by the two methods were consistent taxonomically. In the host prediction of seven other phage contigs by CRISPR spacer similarity alone, six including the five crAssphages had similarity to CRISPR spacers in the genomes of *Bacteroides* and *Porphyromonas*, both of which belong to the order *Bacteroidales*, in at least two datasets. The host for one phage (apr34_1792) was predicted to be *Bifidobacterium* in only the IGCJ dataset (Additional file 2: Table S10). Overall, hosts for 50 plasmid and 11 phage CCs were predicted, while no host was predicted for 21 plasmid CCs by the methods used. In this host prediction, we cannot exclude the possibility that hosts of eMGEs can also be extended to phylogenetically different taxa close to the predicted tax.

### Quantification of gut eMGEs using 413 metagenomic datasets from five countries

**Fig. 3:**
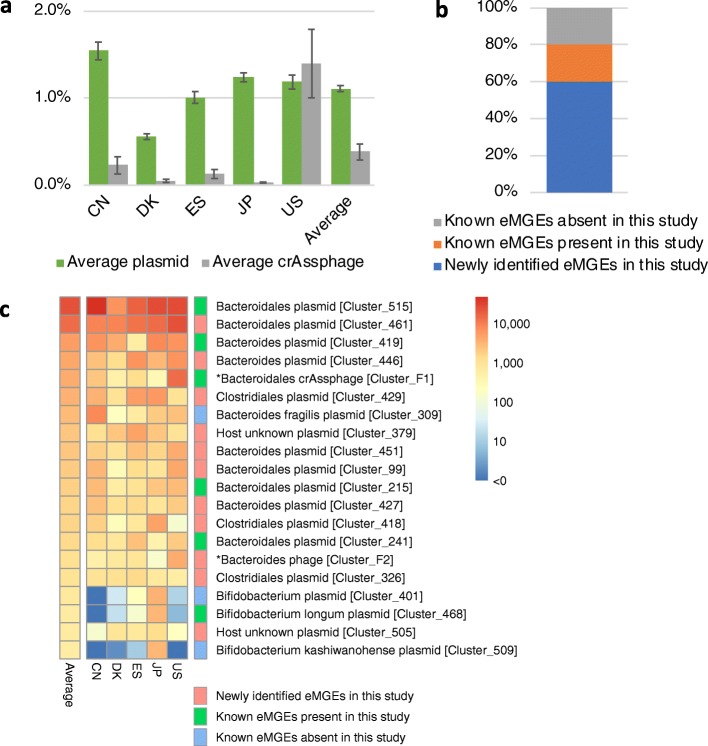
Quantitative analysis of eMGEs in the IGCJ dataset. **a** Average ratios of metagenomic reads mapped to non-redundant eMGE clusters. Error bars represent standard mean errors. **b** Average ratios of reads mapped to three classes of eMGEs. Newly identified eMGEs, known eMGEs present, and known eMGEs absent in this study are shown by blue, orange, and grey, respectively. **c** Heatmap of the abundance of eMGE clusters in the IGCJ dataset. The abundance is the number of reads mapped to eMGEs normalised by length. Colour shades show the degree of abundance of the eMGEs; red indicates relatively high abundance, while blue indicates relatively low abundance. Three classes of eMGEs are also shown by three colours, blue, green, and red, respectively (also see Additional file 2: Table S10)

For quantification of gut eMGEs in the IGCJ dataset, we constructed and used eMGE clusters composed of 563 plasmid and seven phage clusters to which the IGCJ metagenomic reads were mapped. For construction of the eMGE clusters, we first mapped all the plasmids publicly available by IGCJ metagenomic reads with a ≥ 95% identity and excluded the plasmids with mapped coverages < 60% because many of them included plasmids unevenly mapped by non-specific reads containing conserved genes such as transposases and very low-abundance plasmids that were considered to be negligible for quantification. Clustering of the plasmids with mapping coverages ≥ 60%, 11 phage CCs and all publicly available crAssphages generated the eMGE clusters, each of which was composed of highly homologous eMGEs with a ≥ 90% identity and ≥ 70% alignment coverage. Mapping of 10 million (M) short reads per sample to these eMGE clusters revealed that ~ 1.1% of the total reads on average were mapped to the plasmid clusters and ~ 0.38% to the crAssphage cluster (Fig. 3a and Additional file 2: Table S11). Our novel plasmid CCs accounted for ~ 60% of the total reads mapped to the plasmid clusters, indicating that many of them were highly abundant in the IGCJ dataset (Fig. 3b). The inter-country variability in the average abundance of crAssphages (0.03 to 1.4%) was remarkable compared with that of plasmids (0.56 to 1.54%) (Fig. 3a, Additional file 1: Figure S12a, and Additional file 2: Table S11). The increased abundance of crAssphages in the US dataset was largely due to the existence of several subjects having extremely high-abundance crAssphages (up to ~ 21%) but not due to extensive prevalence (Additional file 1: Figure S12b and c). Indeed, the proportion of crAssphage-positive subjects in the US dataset was ~ 53%, slightly lower than the average (~ 60%) of the five countries (Additional file 1: Figure S12c).

**Fig. 4:**
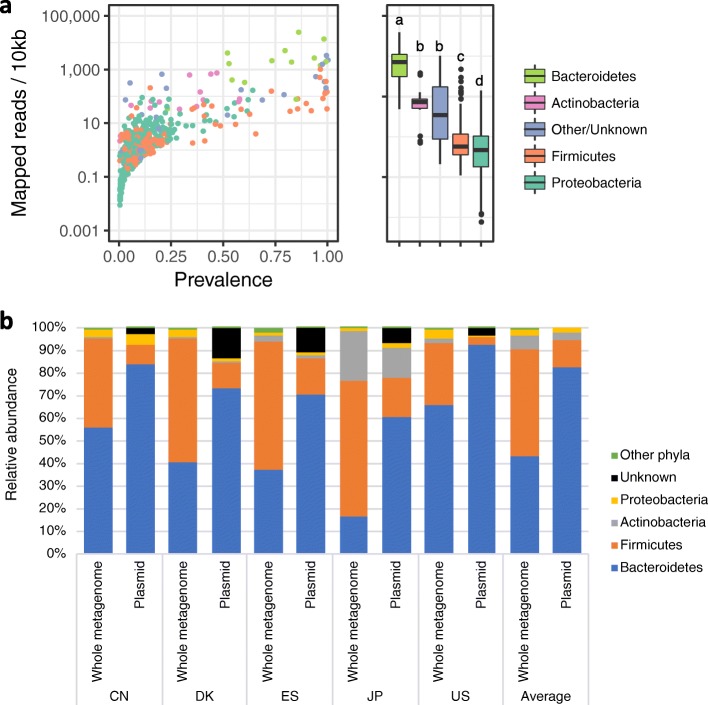
Taxonomic distribution of plasmid-associated hosts in the IGCJ dataset. **a** Abundance and prevalence of plasmid-associated hosts at the phylum level in the IGCJ dataset. The left dot plot shows the abundance (*y*-axis) and the prevalence (*x*-axis) of each plasmid. Putative hosts are assigned to plasmids and are grouped into four major taxa, and unknown and other taxa are coloured differently. The right box plot shows the abundance distributions of the phylum of plasmid hosts depicted by the inter-quartile range (IQR) and median. The whiskers show the lowest and highest values within 1.5 times the IQR. The letters (a, b, c, and d) above the boxes indicate statistically significant (*P* < 0.01) differences between phyla with different letters. **b** Phylum-level compositions of host taxa from the whole metagenome data and plasmids in the five countries. The average abundance of the phyla, depicted by different colours, in the five countries is shown

In the top 20 highly abundant eMGE clusters, 12 including the top four plasmid clusters were associated with Bacteroidetes as putative hosts (Fig. 3c). Likewise, analysis of the host taxon distribution of plasmids revealed that Bacteroidetes-associated plasmids had higher abundance than plasmids associated with other phyla (Fig. 4a). This Bacteroidetes dominance was observed in all the countries, varying from a minimum of 61% in the JP dataset, with 17% Bacteroidetes, to a maximum of 93% in the US dataset, with 66% of the total microbial abundance representing Bacteroidetes (Fig. 4b). The top 20 eMGE clusters included two phage clusters (crAssphage [Cluster_F1] and Bacteroides phage [Cluster_F2]). Notably, the latter (FAKO05_000032F) had higher average mapped reads than the crAssphages in the DK dataset and slightly higher average prevalence (~ 71%) than the crAssphages (~ 60%) in the IGCJ dataset (Additional file 2: Table S11).

We next estimated the ratio of gut plasmids and crAssphages to microbial cells for each of the five countries. The estimation was based on the number of reads mapped to the plasmid and crAssphage clusters and the average sizes of microbial chromosomes, plasmids, and crAssphages. The results revealed that the average ratio of eMGEs to microbial chromosomes ranged from 1.2 to 4.3 for plasmids (3.0 on average) and from 0.01 to 0.7 for crAssphages (0.18 on average) (Additional file 2: Table S12). These data showed that gut plasmids outnumbered microbial cells on average, but crAssphages did not outnumber the microbial cells in the IGCJ dataset. Only in the US dataset were crAssphages close in number to microbial cells, with an average ratio of 0.69. There was no significant correlation between the abundance of crAssphages and subjects’ age, BMI, and sex (Additional file 1: Figure S13).

### Functional profiles of gut plasmids in 413 metagenomic datasets

**Fig. 5:**
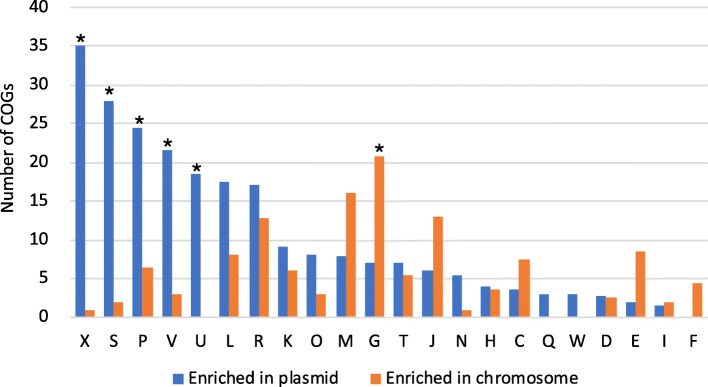
Comparison of COG categories between plasmids and chromosomes. The frequency of COGs (clusters of orthologous groups) is compared between 315 relatively high-abundance plasmid clusters and 249 chromosomes (≥ 0.1% average abundance) in the IGCJ dataset. COG categories with significant differences in enrichment between plasmids and chromosomes are marked with asterisks (*p* < 0.05, Fisher’s exact test). Biological functions are abbreviated by letters; X: Mobilome: prophages, transposons; S: function unknown; P: inorganic ion transport and metabolism; V: defence mechanisms; U: intracellular trafficking, secretion, and vesicular transport; L: replication, recombination and repair; R: general function prediction only; K: transcription; O: posttranslational modification, protein turnover, chaperones; M: cell wall/membrane/envelope biogenesis; G: carbohydrate transport and metabolism; T: signal transduction mechanisms; J: translation, ribosomal structure and biogenesis; N: cell motility; H: coenzyme transport and metabolism; C: energy production and conversion; Q: secondary metabolite biogenesis; W: extracellular structures; D: cell cycle control, cell division, chromosome partitioning; E: amino acid transport and metabolism; I: lipid transport and metabolism, transport and catabolism; F: nucleotide transport and metabolism

Functional annotation of 315 plasmids and 249 chromosomes relatively abundant in the IGCJ dataset revealed that 360 COGs had significant differences (*Q* values < 0.05) in abundance between them, and 233 COGs were significantly enriched in plasmids (Additional file 2: Table S13, see the “Methods” section). In particular, eight were detected only in the plasmids; two were related to inorganic ion transport (COG4264 and COG2370), one was a type IV secretory pathway VirB6 component (COG3704), and the remaining five were uncharacterized. At the higher category level, functions related to the mobilome, including transposase; inorganic ion metabolism, such as iron, cadmium, and copper; defence mechanisms, including restriction-modification, efflux pump, and toxin-antitoxin module; and secretion, such as the type IV secretory pathway, were significantly enriched in the plasmids compared with the chromosomes (*p* < 0.05, Fisher’s exact test). In contrast, functions involved in carbohydrate metabolism were significantly higher (*p* < 0.05) in the chromosomes than in the plasmids (Fig. 5 and Additional file 2: Table S13).

**Fig. 6:**
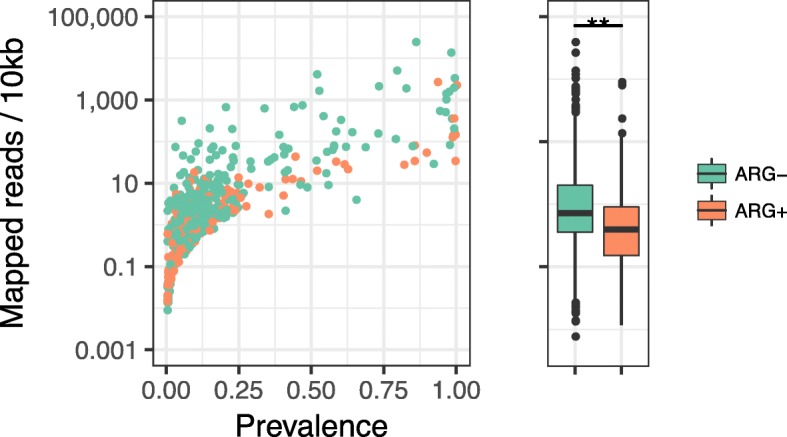
Analysis of antibiotic resistance genes (ARGs) in plasmids. The left dot plot shows the prevalence and abundance of 86 ARG-positive (Additional file 2: Table S14) and 229 ARG-negative plasmids according to the Resfams database. The *y*-axis shows the number of mapped reads per 10-kb region on a log scale. The ARG-positive and ARG-negative plasmids are coloured orange and green, respectively. The right box plot shows the abundance distributions of plasmids with or without ARGs, and their difference is significant as ** denotes *p* < 0.01 (Wilcoxon’s rank-sum test)

We further investigated antibiotic resistance genes (ARGs) using the Resfams database [39] and found that a total of 86 plasmids, including four novel plasmid CCs, were positive for ARG-related genes (Additional file 2: Table S14). Many of the hosts were Proteobacteria, accounting for ~ 76% of the ARG-positive plasmids, Firmicutes with ~ 20%, and a very few Bacteroidetes, but no plasmid was associated with Actinobacteria (Fig. 2a, and Additional file 1: Figure S14a). The frequency of ARGs was similar between the plasmids and chromosomes of Proteobacteria and Firmicutes but lower in the plasmids than in the chromosomes of Bacteroidetes (Additional file 1: Figure S14b). A comparison of ARG-positive and ARG-negative plasmids found that ARGs were more frequently encoded by lower-abundance plasmids (*p* = 2.1e−08, Wilcoxon rank-sum test, Fig. 6). Overall, the present study found several specific functions more frequently harboured by plasmids than by chromosomes in the IGCJ dataset.

## Discussion and conclusions

The present study demonstrated that long-read metagenomic sequencing was useful for the identification of eMGEs as complete contigs and for the exploration of plasmidome entities in the human gut. The plasmid CCs identified by long-read metagenomics included several highly homologous but distinct plasmids, which were hard to distinguish by standard short-read metagenomics. This outcome may be the typical case for insufficient assembly of short reads in the metagenomics of communities containing highly similar sequences longer than the read length. The efficient and accurate reconstruction of eMGEs by long-read metagenomics was achieved by two major steps: we first assembled long reads into contigs using the FALCON assembler, which was originally developed for the assembly of diploid genomes with structural variations without dividing contigs, in a more conservative manner [40], and then processed the assembled contigs with the output binning results of the contigs (see the “Methods” section). Additionally, a remarkable characteristic of the present approach is its ability to identify relatively high-abundance gut eMGEs independent of their sizes, as demonstrated by the reconstruction of two large plasmid CCs with > 600 kb, thereby resulting in the efficient discovery of many novel eMGEs (64/82, 78%).

The 82 CCs were classified as 71 plasmids and 11 phages using several classification assessments (Additional file 2: Table S4). However, one plasmid CC (FA1-2_000589F in Cluster_256) hit a viral contig shorter than the CC, and four homologous plasmid CCs in Cluster_461, which were plasmid-positive by PlasFlow and had partial similarity to a known plasmid pBFUK1, hit several viral contigs. Considering the relatively high abundance of these CCs and the lack of typical structural characteristics of prophages in these CCs, these discrepancies could be explained by contamination of non-viral DNA in the VLPs; hence, these CCs are likely to represent plasmids.

The mapping analysis of IGCJ metagenomic reads showed that the ratio of novel eMGEs was ~ 60%, more than twice the coverage (~ 20%) of known eMGEs alone (Fig. 3b). As described above, because we excluded the plasmids unevenly mapped by non-specific reads from quantification, the observed coverage of the three types of eMGEs may be slightly affected by potential overestimation based on shared genes. The analysis also revealed low coverage of the known plasmid clusters alone, although they represented a large proportion of the plasmid clusters (509/563, 90%). This is probably because they are composed mostly of the plasmids of Proteobacteria species with relatively low abundance in the human gut. In other words, the present study efficiently identified many plasmids hitherto unknown but abundant in the human gut.

It was reported that crAssphages were identified as circular genomes [31, 41]. However, our analysis provided evidence suggesting that the five crAssphages had linear genomes with TDRs (Fig. 2 and Additional file 1: Figure S5). In a previous study, a circular crAssphage genome was validated by gap closing between fragmented contigs by PCR, followed by sequencing of PCR products [31]. However, PCR amplification between unconnected TDRs in the linear genome is also feasible by duplex formation via annealing between downstream TDRs in the extended DNAs primed from the flanking regions of TDRs, similar to the mechanism for extended primer dimer formation or template switching [42], although we cannot exclude the possibility of coexistence of both circular and linear crAssphage genomes.

Although crAssphages were also reported to be highly abundant in the human gut, the ratio of mapped reads varied from 0.03% (JP) to 1.4% (US) among the five countries (Fig. 3 and Additional file 1: Figure S12). In addition, the proportion of crAssphage-positive subjects was as low as 60% on average in the 413 individuals (Additional file 1: Figure S12). These data suggest high variability in crAssphages at both the individual and country levels and the presence of two types of gut microbiomes: those with high and low abundance of crAssphages. However, we could not link the abundance and prevalence of crAssphages to the overall microbial composition or the host’s genetic background, age, BMI, and sex (Additional file 1: Figure S13). There are several questions that arise from these data. For example, what is the real role of crAssphages in the gut ecosystem? and what is the factor affecting this dominant phage?

The ratio of plasmids to microbial chromosomes in the human gut metagenome has not previously been reported. Our first estimation suggested that plasmids outnumber the microbial cells in IGCJ gut microbiomes. On the other hand, the estimated ratio of crAssphages to microbial cells is approximately consistent with previous estimations of gut phages to microbial cells, ranging from 0.1:1 to 1:1 [6, 43]. The present estimate remains tentative because yet-unidentified eMGEs should exist and will need to be confirmed with more samples.

Host prediction is a challenging issue in eMGE study [9, 38]. A similarity search for the draft genomes of individual cultured species containing unidentified plasmid sequences is a simple but solid method for host assignment of plasmids, once plasmids are identified as complete CCs. Indeed, in this study, hosts for 36 of the 71 plasmid CCs were assigned by a similarity search for draft genomes, of which 13 hosts were also predicted by CO and/or MM to taxonomically close species assigned by the similarity search. In addition, the hosts of two plasmid CCs predicted by both CO and MM and those of four phage CCs predicted by both MM and CRISPR spacer were taxonomically consistent between the two methods (Additional file 2: Tables S9 and S10). Thus, there was almost no inconsistency in host prediction between at least two different methods, and many of the predicted hosts were taxonomically assigned at the species and genus levels, demonstrating the practical usefulness of the three methods and their combined use for host prediction of eMGEs, as well as the Hi-C method recently developed [44]. In addition, the overall sequence similarity shown here could also be a useful index for host prediction of plasmids, because plasmids from taxonomically similar hosts tended to have relatively high sequence similarities between them (Fig. 2a).

In host prediction of phages, YS1-2_2434 and FAKO27_000271F may be novel phages of putative hosts *Bifidobacterium* and *Faecalibacterium*, respectively, because they differed from the recently reported prophages of these two taxa [45, 46]. FAKO27_000238F may also be a novel phage and the first associated with *Phascolarctobacterium* as a putative host.

The present analysis also revealed the largest host-plasmid network and the highest abundance of plasmids in Bacteroidetes, which was nearly independent of the overall microbial composition. These results may accord with the previous findings that there was no profound association between the dominant species and its mobile genes and the extensive DNA transfer between *Bacteroidales* species in the human gut [8, 47]. Taken together, our data strongly suggest that Bacteroidetes-associated plasmids are the major players and mediators in modulating human gut microbiome structure and function toward improving the adaptability of the host to environmental changes such as an increase in heavy metal ions.

The functional analysis identified several plasmid-enriched functions, such as transposase, toxin-antitoxin, type IV secretion system (conjugation), and inorganic ion transport (Fig. 5 and Additional file 2: Table S13). Among the genes in category X, transposase-related COGs were exclusively identified as plasmid-enriched genes, which may be partly because category X is biased toward many transposases in its composition. While the former three functions were known to be plasmid-enriched [48, 49], we also found the dissemination of resistance and efflux systems for metal ions such as copper, arsine, tellurium, and cadmium in gut plasmids, suggesting that gut plasmids are determinants of metabolism for toxic metal ions [50]. Our data also revealed that antibiotic functions were strongly linked to relatively low-abundance Proteobacteria plasmids, particularly *Enterobacteriaceae*, in the human gut (Fig. 2a and Additional file 2: Table S14), suggesting associations between nosocomial *Enterobacteriaceae* species and the human gut microbiome [51]. However, at present, we do not know the biological significance of the tendency to carry plasmids encoding antibiotic functions more frequently in low-abundance species than high-abundance plasmids.

In conclusion, long-read metagenomics provides an efficient method for the exploration of uncharted eMGEs in the human gut, and the accumulated data represent an alternative resource useful for a deeper understanding of human gut microbial ecology.

## Methods

### Subjects, samples, and faecal DNA preparations

We recruited 12 Japanese volunteers, of whom six subjects were the same as those who donated faecal samples in a previous study [20] and six other subjects were members of a family: two parents, two children, and two grandparents. No subjects were treated with antibiotics during faecal sample collection.

Faecal samples were transferred under anaerobic conditions at 4 °C to the laboratory within 36 h after defecation, immediately frozen with liquid nitrogen, and stored at − 80 °C until use. We collected 13 faecal samples from the 12 individuals, including a second sample (biological replicate) from an individual (denoted by ES) 2 months after the collection of the first sample. High-molecular-weight DNA samples were prepared by the enzymatic lysis method [52, 53]. Prior to DNA extraction, each faecal sample suspended in PBS buffer was filtered with a 100-μm-mesh nylon filter (Corning Inc., New York, NY, USA) to remove human and eukaryotic cells and other debris from the faecal sample. The debris on the filter was washed twice using a glass or plastic bar with PBS buffer. The bacteria-enriched pellet was obtained by centrifugation of the filtrate at 5000 rpm for 10 min at 4 °C [53].

### Sequencing of faecal DNA samples

For SMRTbell library preparation, faecal DNA was sheared using a g-TUBE device (Covaris Inc., Woburn, MA, USA) at 4300 rpm and purified using a 0.45× volume ratio of AMPure beads (Pacific Biosciences, Menlo Park, CA, USA). SMRTbell libraries for sequencing were prepared using the “20-kb Template Preparation using BluePippin™ Size Selection System (15-kb Size Cutoff)” protocol. Briefly, the steps included (1) DNA repair, (2) blunt ligation with hairpin adapters with the SMRTbell template Prep Kit 1.0 (Pacific Biosciences), (3) 7-kb size cutoff size selection using the BluePippin DNA Size Selection System by Sage Science, and (4) binding to polymerase P6 using DNA Sequencing Reagent 4.0 (Pacific Biosciences). SMRTbell libraries were sequenced on SMRT Cells (Pacific Biosciences) using magnetic bead loading and P4-C2 or P6-C4 chemistry. Sequence data were collected according to the magnetic bead collection protocol, 10-kb insert size, stage start, and 360-min movies in PacBio RS Remote. Primary filtering was performed on the PacBio RS II Blade Center server. The sequences mapped to the human genome (hg19) were removed prior to submission of PacBio reads to the NCBI Sequence Read Archive (SRA) using DAMAPPER (https://github.com/thegenemyers/DAMAPPER), a modified version of DALIGNER [54].

For short-read sequencing of seven newly collected samples in this study with the MiSeq platform, DNA libraries were prepared using the SPARK DNA sample Prep Kit (Qiagen, Beverly, MA, USA). Quality control of the metagenomic reads was conducted as described previously [20]. Briefly, low-quality bases and reads were filtered using the FASTX tool kit (http://hannonlab.cshl.edu/fastx_toolkit/). Host-derived reads were excluded by mapping the reads to the reference human genome (hg19) using Bowtie2 (v.2.2.1) software [55]. The ratio of reads mapped to the human genome was < 0.1% in both the long- and short-read sequencing (Additional file 2: Table S1). The very low ratio of human reads in our metagenomic data can be explained by the efficient removal of human cells from the faecal samples by filtration prior to DNA extraction [53], as described above. Additional metagenomic short reads (Roche 454, Ion PGM, and Illumina MiSeq) publicly available from the five countries [19, 20] were downloaded from the NCBI SRA.

### Assembly of PacBio reads and short reads

For assembly of the PacBio metagenomic reads, we used FALCON v0.2 software (https://github.com/PacificBiosciences/FALCON) [40]. Because FALCON tended to extend contigs to merge DNA sequences from distinct microbial species to generate erroneous contigs, we used unitigs, basic blocks of contigs that are shorter but more reliable contiguous sequences than contigs.

To reconstruct circular contigs after FALCON assembly, we used the binning results of MetaBAT [56] as external guiding information with a single criterion: if a node in the assembly graph had only one in-edge and one out-edge that belonged to the same MetaBAT bin ID, then we merged the two edges representing unitigs to generate circular contigs. Note that a distinct bin ID was assigned to each unbinned unitig to avoid self-loops in the graph. This is the first attempt to map external binning information onto an assembly graph to untangle chimeric nodes in the graph. This method achieved reliable elongation of contigs by using the binning information to produce a more conservative layout of contigs than the original FALCON assembly did. To reconstruct relatively small circular contigs representing eMGEs, we used the cutoff values 2000 bp and 2200 bp for overlaps between raw subreads and between error-corrected subreads (technically, “preads”), respectively. These parameters influence the minimum length of the CCs generated by the assembly. After polishing the contigs with long reads using Quiver from the SMRT Pipe (v.1.87) software, the standard pipeline provided by Pacific Biosciences, we further corrected errors in the contigs using Pilon (v.1.12) [57], a software for error correction by short reads. The read depth of the assembled contigs was determined by PacBio’s standard software. De novo assembly of the metagenomic short reads (Roche 454, Ion PGM, and Illumina MiSeq) was performed by MEGAHIT (v1.1.1) [58].

### Alignment of PacBio and short-read contigs

PacBio and short-read contigs were aligned using NUCmer (v3.1) software. Alignments with length coverage < 95% or sequence similarity < 95% were removed, and then, the sequence similarity of the alignments was calculated.

### Estimation of microbial composition from PacBio and MiSeq data

To obtain the microbial composition from the PacBio data, we first predicted protein-coding genes in the PacBio contigs using Prodigal software [59]. The genes were aligned to the 6149 reference genomes [20] using BLASTN with a > 95% identity and > 90% length coverage to assign the taxa [60]. The relative abundance of the genomes/taxa was calculated by counting the number of genes aligned, multiplying the number of genes by the read depth of the contig, and normalising by gene length. Estimation of microbial composition from the MiSeq data was conducted by mapping the reads to the reference genomes using Bowtie2 with a 95% identity threshold and normalising the number of mapped reads by genome size [20]. The similarity between the microbial compositions obtained from PacBio and MiSeq data was assessed with Pearson’s correlation coefficient.

### Classification of CCs as plasmids and phages

In the classification assessment using POGs [29], we determined the threshold of identity and length coverage to perform the highest confidence (Additional file 1: Figure S2) using reference phages (*n* = 1957) as positive data and reference plasmids (*n* = 6589) as negative data available from NCBI on June 2016. By aligning the genes to POGs with BLASTP, the threshold (> 90% length coverage) for classification of CCs as phages was determined. For classification of CCs as phages, VirSorter (v1.0.3) [30] was also employed with the virome database and default options in the CyVerse environment [61]. Categories 1, 2, 4, and 5 were considered to classify CCs as phages, while categories 3 and 6 were excluded because these categories included false positives [62]. PlasFlow (v1.1) was used with the default options for classification of CCs as plasmids [30].

Functional annotation of genes in the CCs was conducted using Prokka [63] and the COG database (BLASTP with the *e* value < 0.00001). The presence and absence of known plasmid-enriched COGs related to plasmid replication, toxin-antitoxin system, and type IV secretion system (COG1475, COG2026, COG2126, COG2336, COG2948, COG3077, COG3451, COG3505, COG3704, COG3736, COG3843, COG5527, and COG5655) were investigated for CCs.

A similarity search of CCs for the public plasmid/phage database and phage sequences in the IMG/VR [33] and VirSorter [29] databases was conducted using NUCmer [64], in which CCs with sequence similarity ≥ 90% and length coverage ≥ 70% to the references were assigned to the corresponding plasmids and phages, respectively.

The whole sequence comparison of the 71 plasmid CCs and 114 known/reference plasmids relatively abundant in the human gut was performed using TBLASTX [65]. The 114 known/reference plasmids used in this analysis had average mapped reads of > 5 per kb in the IGCJ dataset. The obtained dendrogram was visualised using iTOL software [66].

### Analysis of crAssphage genomes

PacBio subreads and MiSeq reads were aligned to the five CCs assigned to crAssphage. To assess the alignments, they were visualised using IGV [67]. The sequences of the terminal direct repeats (TDRs) of the five CCs were obtained by reassembling subreads starting/ending at either side of the TDRs. MiSeq reads were further aligned to the TDR sequences using Bowtie2 to manually determine the exact ends of TDRs. To convert the circular genome of the crAssphage (NC_024711) in GenBank [31] to a linear genome, the TDRs were determined by aligning the TDRs of the five crAssphage CCs to the circular genome with BLASTN. Protein-coding genes in the linear crAssphage genomes were predicted using MetaGeneMark [68], and the conserved genes in the six crAssphage genomes were investigated using Roary software [69] with the “-p 80” option. The structures of the six crAssphage genomes were visualised using the genoPlotR package [70] in R software and custom Perl scripts. GC skew was calculated for a 100-bp sliding window with a 50-bp step size.

### Quantification of eMGEs including the 82 CCs in the IGCJ dataset

We obtained all metagenomic reads from a total of 413 healthy faecal samples of Japanese (*n* = 106) [20], Danish (*n* = 84) and Spanish (*n* = 59) [3, 19, 71], American (*n* = 90) [4], and Chinese (*n* = 74) [72] people from http://public.genomics.org.cn, HMP DACC (http://www.hmpdacc.org), and/or the NCBI SRA to construct the IGCJ dataset. This dataset did not include data from patients with inflammatory bowel disease and type 2 diabetes. The metagenomic reads in the IGCJ dataset were subjected to quality control under the same conditions as described previously [20].

The eMGE clusters composed of 563 plasmid and seven phage clusters were constructed as follows. The IGCJ metagenomic reads (10 M reads per sample) were first mapped to all the publicly available plasmids and the 71 plasmid CCs using Bowtie2 with a 95% identity threshold. The reads hit > 3000 plasmids, from which plasmids with map coverages < 60% were excluded (see the “Results” section). The 1162 plasmids with mapped coverages ≥ 60% were then clustered with a ≥ 90% identity, ≥ 70% alignment coverage, and ≥ 0.7 ratio of shorter to longer sequences using NUCmer to generate 563 plasmid clusters. The breakdown of the plasmid clusters was 509 clusters of known/reference plasmids alone, 47 clusters of the novel plasmid CCs alone, and seven clusters of both plasmid CCs and homologous known plasmids (Additional file 2: Table S11). Similarly, we obtained a cluster of crAssphages and six unique clusters from the 11 phage CCs. The mapping of 10 M metagenomic reads per sample to the eMGE clusters was conducted with a ≥ 95% identity. The number of reads mapped to the clusters was normalised to the length of the longest representative eMGE in the cluster.

### Host prediction of eMGEs

For host assignment of plasmids by similarity search, plasmid CCs were aligned to 5353 draft genomes publicly available with NUCmer [64], and draft genomes having a ≥ 90% identity and ≥ 70% length coverage with the CCs were assigned as the hosts of the corresponding plasmids.

For co-occurrence (CO) analysis, we mapped metagenomic reads of the IGCJ dataset to reference genomes and eMGEs with a 95% identity threshold to obtain the abundance normalised by genome size. Spearman’s correlation coefficients (SCCs) were then calculated for variance in the abundance of chromosomes and eMGEs across the samples, and the genomes having SCCs of ≥ 0.7 with the eMGEs were predicted to be putative hosts of the corresponding eMGEs.

For host prediction of phages by CRISPR spacer similarity, we used three datasets of host genomes: the public genome database, contigs with ≥ 500 bp generated from assembly of metagenomic reads in the IGCJ dataset using MEGAHIT (v1.1.1) [58], and contigs generated from the assembly of PacBio subreads in the JP PacBio dataset using Pilercr (version 1.06) [73]. CRISPR spacers (≥ 20 bp) in microbial genomes and contigs were detected using Pilercr with the default options. The detected CRISPR spacers were aligned to the phage genomes using BLASTN with the following options: -e 1 –G 10 –E 2 –q 1 –W 7 –F F; this served to identify microbial genomes and contigs containing CRISPR spacers with 0 or one mismatch and > 95% alignment coverage between them. The microbial taxa of the genomes and contigs were determined by their alignment using NUCmer to the reference genomes with a ≥ 90% identity and ≥ 50% alignment coverage.

The PacBio SMRT system can detect modified bases, such as 6-methyladenine (m6A) and 4-methylcytosine (m4C), because inter-pulse duration (IPD) between neighbouring bases is likely to be longer when the first bases are modified [65], and the modification is detectable by monitoring the IPD ratios of modified bases to those of unmodified ones. According to the process described previously [27], we first determined the optimal parameters of “methylation fraction” (percentage of motif sequences methylated), “mean coverage” (average sequencing read-depth per strand on the motif sites), and “mean IPD ratio” to 0.6, 25, and 2.5 as the thresholds, respectively, from PacBio reads from a mock community composed of eight bacteria with and without plasmids (*Lactobacillus paralimentarius* JCM 10707, *Natronolimnobius baerhuensis* JCM 12253, *Bacillus cereus* ATCC 14579, *Variovorax* sp. JCM 16519, *Clostridiales* bacterium ACSP 3, *Staphylococcus aureus* HSAU10, *Bifidobacterium longum* IBLI, and *Escherichia coli* SE11). We then filtered for methylation motifs (MMs) in the HQ chromosome bins with the optimised methylation fraction and mean coverage. In this process, we excluded the motif G^m6^ATC from host prediction because this motif was ubiquitous among bacteria. Using the filter-passed chromosomal MMs as baits, we calculated the mean IPD ratio values of the MMs in each eMGE and HQ chromosome bin and binarized the values according to the threshold (i.e., IPD ratios higher than the threshold were defined as 1 to indicate methylation, and the others were defined as 0 to indicate nonmethylation). Finally, we linked the eMGEs and the HQ chromosome bins, between which at least one MM was shared, and the binarized IPD ratio values were equivalent except missing values.

The results of host prediction of the plasmid CCs were summarised and visualised as a host-plasmid network using Cytoscape. In this analysis, taxonomically undefined bacterial species (e.g., *Bacteroides* sp.) were changed to taxonomically defined bacterial species of which the 16S rRNA gene sequence had ≥ 99.8% identity with that of the undefined species.

### Comparison of functions between plasmids and chromosomes

For comparison of the frequency of COGs between plasmids and chromosomes, we used 315 relatively abundant plasmids (≥ 1 average mapped reads per 10 kb) and complete chromosomes of 249 microbial species with ≥ 0.1% average abundance in the IGCJ dataset. The genes were functionally annotated by BLASTP to the COG database with the *e* value < 0.00001 using Prodigal [59]. Statistical significance was calculated using Fisher’s exact test, and *p* values were transformed to *q* values [74]. Antibiotic resistance genes were identified by searching Resfams database [39] using the hmmscan function of HMMER3 [75] with the gathering thresholds. The abundances of the ARG-positive and ARG-negative plasmids were compared using the Wilcoxon rank-sum test.

### Reconstruction and analysis of HQ chromosome bins

For reconstruction of chromosome bins from the PacBio contigs in the 12 JP samples, metagenomic short reads (10 M reads per sample) of 106 JP individuals [20] were mapped to PacBio contigs by Bowtie2. Based on read depth and tetranucleotide frequency, contigs were clustered to chromosome bins using MetaBAT (v.0.26.3) [56] with the “--minMapQual 4 --verysensitive” options. The completeness and contamination were calculated by the presence or absence of single-copy marker genes using CheckM (v.1.0.5) [76], and high-quality (HQ) chromosome bins with > 90% completeness and < 5% contamination were defined. We deposited the sequences of 101 HQ chromosome bins tagged with the ‘long-read metagenome-assembled genome (LMAG)’ in a public database (Additional file 2: Table S7).

Taxonomic assignment of the HQ chromosome bins was conducted as previously described [36]. Briefly, the protein-coding genes predicted by Prodigal were aligned to 40 single-copy marker genes using BLASTP with an *e* value < 0.00001. The marker genes identified in the HQ chromosome bins were then aligned to those of the reference genomes using glsearch (v.36.3.5e) [77]. The HQ chromosome bins having length-weighted average identity ≥ 96.5% with the reference genomes were assigned the same taxa as the reference genomes.

The phylogenetic tree of 101 HQ chromosome bins and 181 reference genomes with ≥ 0.05% relative abundance in the 12 subjects was constructed based on the similarity of amino acid sequences of the 40 marker genes using the neighbour-joining method in MEGA (v.6.06) [78] and visualised with iTOL [66]. The similarities of the marker genes were calculated by MAFFT (v.7.043b) [79] with the “--localpair --maxiterate 1000” options.

## Additional files

Additional file 1: **Figure S1.** Genes in PacBio and short-read contigs. **Figure S2.** Optimization for identification of phage orthologous groups (POGs). **Figure S3.** Sequence alignments of two highly homologous but distinct plasmid CCs in three samples. **Figure S4.** Similarity search of 82 CCs against the public plasmid/phage database. **Figure S5.** Mapping of PacBio subreads and short reads to the five crAssphage CCs. **Figure S6.** Dot plot of terminal direct repeats in the five crAssphages. **Figure S7.** GC skews in the linear crAssphage genomes. **Figure S8.** Mapping of PacBio subreads to two phage CCs. **Figure S9.** Phylogenetic tree of 101 high-quality chromosome bins and 181 known genomes. **Figure S10.** Host prediction by methylation motif similarity between eMGEs and HQ chromosome bins in the PacBio JP dataset. **Figure S11.** Host-plasmid network. The predicted host-plasmid relationships were summarized and visualized as a network. **Figure S12.** Ratios of reads mapped to plasmids and crAssphages in 413 metagenomic data sets and proportions of crAssphage-positive individuals. **Figure S13.** Association analysis of the abundance of crAssphages with subjects’ age, BMI, and sex in the IGCJ dataset. **Figure S14.** Antibiotic resistance genes in plasmids in the IGCJ dataset. (PDF 6178 kb)

Additional file 2: **Table S1.** Summary of metagenomic sequencing of fecal samples from 12 individuals by PacBio and other sequencers. **Table S2.** Contigs generated from assembly of PacBio and short reads. **Table S3.** Distribution of read-depths and contig lengths in PacBio contigs of the three subjects. **Table S4.** Classification and characterization of 82 circular contigs <1-Mb in PacBio assembly. **Table S5.** Functional annotation of the 71 plasmid CCs based on COGs. **Table S6.** Intra-similarity and length of terminal direct repeats (TDRs) of crAssphage linear genomes. **Table S7.** High-quality chromosome bins reconstructed from PacBio contigs. **Table S8.** Host prediction by similarity search of the 71 plasmid CCs for the public genome database. **Table S9.** Summary of host prediction of the 71 plasmid CCs. **Table S10.** Summary of host prediction of the 11 phage CCs. **Table S11.** Clusters of plasmids and phages, putative hosts, and the number of reads mapped to the clusters in the IGCJ dataset. **Table S12.** Estimation of ratio of plasmids and crAssphage per microbial chromosome in the IGCJ dataset. **Table S13.** COGs having significant difference in abundance between plasmids and reference genomes detected in the IGCJ dataset. **Table S14.** Resfams-based antibiotic resistance functions in plasmids detected in the IGCJ dataset. (XLSX 221 kb)

## Data Availability

The accession numbers for the sequences of 71 plasmid contigs, 11 phage contigs, and 101 HQ chromosome bins are CP021560-CP021639, MK415399-MK415410, and NAJS00000000-NANO00000000, respectively, and are available from the Sequence Read Archive (SRA) (BioProject SRP098614).

## References

[1] Gill SR, Pop M, Deboy RT, Eckburg PB, Turnbaugh PJ, Samuel BS, Gordon JI, Relman DA, Fraser-Liggett CM, Nelson KE (2006). Metagenomic analysis of the human distal gut microbiome. Science.

[2] Kurokawa K, Itoh T, Kuwahara T, Oshima K, Toh H, Toyoda A, Takami H, Morita H, Sharma VK, Srivastava TP (2007). Comparative metagenomics revealed commonly enriched gene sets in human gut microbiomes. DNA Res.

[3] Qin J, Li R, Raes J, Arumugam M, Burgdorf KS, Manichanh C, Nielsen T, Pons N, Levenez F, Yamada T (2010). A human gut microbial gene catalogue established by metagenomic sequencing. Nature.

[4] Human Microbiome Project C (2012). Structure, function and diversity of the healthy human microbiome. Nature.

[5] Koonin EV, Wolf YI (2008). Genomics of bacteria and archaea: the emerging dynamic view of the prokaryotic world. Nucleic Acids Res.

[6] Reyes A, Semenkovich NP, Whiteson K, Rohwer F, Gordon JI (2012). Going viral: next-generation sequencing applied to phage populations in the human gut. Nat Rev Microbiol.

[7] Virgin HW (2014). The virome in mammalian physiology and disease. Cell.

[8] Brito IL, Yilmaz S, Huang K, Xu L, Jupiter SD, Jenkins AP, Naisilisili W, Tamminen M, Smillie CS, Wortman JR (2016). Mobile genes in the human microbiome are structured from global to individual scales. Nature.

[9] Dib JR, Wagenknecht M, Farias ME, Meinhardt F (2015). Strategies and approaches in plasmidome studies-uncovering plasmid diversity disregarding of linear elements?. Front Microbiol.

[10] Jorgensen TS, Kiil AS, Hansen MA, Sorensen SJ, Hansen LH (2014). Current strategies for mobilome research. Front Microbiol.

[11] Reyes A, Haynes M, Hanson N, Angly FE, Heath AC, Rohwer F, Gordon JI (2010). Viruses in the faecal microbiota of monozygotic twins and their mothers. Nature.

[12] Minot S, Sinha R, Chen J, Li H, Keilbaugh SA, Wu GD, Lewis JD, Bushman FD (2011). The human gut virome: inter-individual variation and dynamic response to diet. Genome Res.

[13] Minot S, Grunberg S, Wu GD, Lewis JD, Bushman FD (2012). Hypervariable loci in the human gut virome. Proc Natl Acad Sci U S A.

[14] Minot S, Bryson A, Chehoud C, Wu GD, Lewis JD, Bushman FD (2013). Rapid evolution of the human gut virome. Proc Natl Acad Sci U S A.

[15] Castro-Mejia JL, Muhammed MK, Kot W, Neve H, Franz CM, Hansen LH, Vogensen FK, Nielsen DS (2015). Optimizing protocols for extraction of bacteriophages prior to metagenomic analyses of phage communities in the human gut. Microbiome.

[16] Manrique P, Bolduc B, Walk ST, van der Oost J, de Vos WM, Young MJ (2016). Healthy human gut phageome. Proc Natl Acad Sci U S A.

[17] Shkoporov AN, Ryan FJ, Draper LA, Forde A, Stockdale SR, Daly KM, McDonnell SA, Nolan JA, Sutton TDS, Dalmasso M (2018). Reproducible protocols for metagenomic analysis of human faecal phageomes. Microbiome.

[18] Nielsen HB, Almeida M, Juncker AS, Rasmussen S, Li J, Sunagawa S, Plichta DR, Gautier L, Pedersen AG, Le Chatelier E (2014). Identification and assembly of genomes and genetic elements in complex metagenomic samples without using reference genomes. Nat Biotechnol.

[19] Li J, Jia H, Cai X, Zhong H, Feng Q, Sunagawa S, Arumugam M, Kultima JR, Prifti E, Nielsen T (2014). An integrated catalog of reference genes in the human gut microbiome. Nat Biotechnol.

[20] Nishijima S, Suda W, Oshima K, Kim SW, Hirose Y, Morita H, Hattori M (2016). The gut microbiome of healthy Japanese and its microbial and functional uniqueness. DNA Res.

[21] Sharon I, Kertesz M, Hug LA, Pushkarev D, Blauwkamp TA, Castelle CJ, Amirebrahimi M, Thomas BC, Burstein D, Tringe SG (2015). Accurate, multi-kb reads resolve complex populations and detect rare microorganisms. Genome Res.

[22] Kuleshov V, Jiang C, Zhou W, Jahanbani F, Batzoglou S, Snyder M (2016). Synthetic long-read sequencing reveals intraspecies diversity in the human microbiome. Nat Biotechnol.

[23] Brown BL, Watson M, Minot SS, Rivera MC, Franklin RB (2017). MinION nanopore sequencing of environmental metagenomes: a synthetic approach. Gigascience.

[24] Frank JA, Pan Y, Tooming-Klunderud A, Eijsink VG, McHardy AC, Nederbragt AJ, Pope PB (2016). Improved metagenome assemblies and taxonomic binning using long-read circular consensus sequence data. Sci Rep.

[25] Tsai YC, Conlan S, Deming C, Program NCS, Segre JA, Kong HH, Korlach J, Oh J (2016). Resolving the complexity of human skin metagenomes using single-molecule sequencing. MBio.

[26] Bishara Alex, Moss Eli L, Kolmogorov Mikhail, Parada Alma E, Weng Ziming, Sidow Arend, Dekas Anne E, Batzoglou Serafim, Bhatt Ami S (2018). High-quality genome sequences of uncultured microbes by assembly of read clouds. Nature Biotechnology.

[27] Beaulaurier J, Zhu S, Deikus G, Mogno I, Zhang XS, Davis-Richardson A, Canepa R, Triplett EW, Faith JJ, Sebra R (2018). Metagenomic binning and association of plasmids with bacterial host genomes using DNA methylation. Nat Biotechnol.

[28] Kristensen DM, Cai X, Mushegian A (2011). Evolutionarily conserved orthologous families in phages are relatively rare in their prokaryotic hosts. J Bacteriol.

[29] Roux S, Enault F, Hurwitz BL, Sullivan MB (2015). VirSorter: mining viral signal from microbial genomic data. PeerJ.

[30] Krawczyk PS, Lipinski L, Dziembowski A (2018). PlasFlow: predicting plasmid sequences in metagenomic data using genome signatures. Nucleic Acids Res.

[31] Dutilh BE, Cassman N, McNair K, Sanchez SE, Silva GG, Boling L, Barr JJ, Speth DR, Seguritan V, Aziz RK (2014). A highly abundant bacteriophage discovered in the unknown sequences of human faecal metagenomes. Nat Commun.

[32] Roux S, Hallam SJ, Woyke T, Sullivan MB. Viral dark matter and virus-host interactions resolved from publicly available microbial genomes. Elife. 2015;4. 10.7554/eLife.08490. 10.7554/eLife.08490 PMC4533152 26200428

[33] Paez-Espino D, Chen IA, Palaniappan K, Ratner A, Chu K, Szeto E, Pillay M, Huang J, Markowitz VM, Nielsen T (2017). IMG/VR: a database of cultured and uncultured DNA viruses and retroviruses. Nucleic Acids Res.

[34] Chung CH, Walter MH, Yang L, Chen SG, Winston V, Thomas MA (2017). Predicting genome terminus sequences of Bacillus cereus-group bacteriophage using next generation sequencing data. BMC Genomics.

[35] Mende DR, Sunagawa S, Zeller G, Bork P (2013). Accurate and universal delineation of prokaryotic species. Nat Methods.

[36] Antipov D, Hartwick N, Shen M, Raiko M, Lapidus A, Pevzner PA (2016). plasmidSPAdes: assembling plasmids from whole genome sequencing data. Bioinformatics.

[37] Stern A, Mick E, Tirosh I, Sagy O, Sorek R (2012). CRISPR targeting reveals a reservoir of common phages associated with the human gut microbiome. Genome Res.

[38] Edwards RA, McNair K, Faust K, Raes J, Dutilh BE (2016). Computational approaches to predict bacteriophage-host relationships. FEMS Microbiol Rev.

[39] Gibson MK, Forsberg KJ, Dantas G (2015). Improved annotation of antibiotic resistance determinants reveals microbial resistomes cluster by ecology. ISME J.

[40] Chin CS, Peluso P, Sedlazeck FJ, Nattestad M, Concepcion GT, Clum A, Dunn C, O'Malley R, Figueroa-Balderas R, Morales-Cruz A (2016). Phased diploid genome assembly with single-molecule real-time sequencing. Nat Methods.

[41] Guerin E, Shkoporov A, Stockdale SR, Clooney AG, Ryan FJ, Sutton TDS, Draper LA, Gonzalez-Tortuero E, Ross RP, Hill C (2018). Biology and taxonomy of crAss-like bacteriophages, the most abundant virus in the human gut. Cell Host Microbe.

[42] Patel R, Lin M, Laney M, Kurn N, Rose S, Ullman EF (1996). Formation of chimeric DNA primer extension products by template switching onto an annealed downstream oligonucleotide. Proc Natl Acad Sci U S A.

[43] Kim MS, Park EJ, Roh SW, Bae JW (2011). Diversity and abundance of single-stranded DNA viruses in human feces. Appl Environ Microbiol.

[44] Stewart RD, Auffret MD, Warr A, Wiser AH, Press MO, Langford KW, Liachko I, Snelling TJ, Dewhurst RJ, Walker AW (2018). Assembly of 913 microbial genomes from metagenomic sequencing of the cow rumen. Nat Commun.

[45] Duranti S, Lugli GA, Mancabelli L, Armanini F, Turroni F, James K, Ferretti P, Gorfer V, Ferrario C, Milani C (2017). Maternal inheritance of bifidobacterial communities and bifidophages in infants through vertical transmission. Microbiome.

[46] Cornuault JK, Petit MA, Mariadassou M, Benevides L, Moncaut E, Langella P, Sokol H, De Paepe M (2018). Phages infecting Faecalibacterium prausnitzii belong to novel viral genera that help to decipher intestinal viromes. Microbiome.

[47] Coyne MJ, Zitomersky NL, McGuire AM, Earl AM, Comstock LE (2014). Evidence of extensive DNA transfer between Bacteroidales species within the human gut. mBio.

[48] Smillie C, Garcillan-Barcia MP, Francia MV, Rocha EP, de la Cruz F (2010). Mobility of plasmids. Microbiol Mol Biol Rev.

[49] Ogilvie LA, Firouzmand S, Jones BV (2012). Evolutionary, ecological and biotechnological perspectives on plasmids resident in the human gut mobile metagenome. Bioeng Bugs.

[50] Silver S, Walderhaug M (1992). Gene regulation of plasmid- and chromosome-determined inorganic ion transport in bacteria. Microbiol Rev.

[51] San Millan A (2018). Evolution of plasmid-mediated antibiotic resistance in the clinical context. Trends Microbiol.

[52] Kim SW, Suda W, Kim S, Oshima K, Fukuda S, Ohno H, Morita H, Hattori M (2013). Robustness of gut microbiota of healthy adults in response to probiotic intervention revealed by high-throughput pyrosequencing. DNA Res.

[53] Ueno M, de Bruijn FJ (2011). Assessment and improvement of methods for microbial DNA preparation from fecal samples. Handbook of molecular microbial ecology II: metagenomics in different habitats.

[54] Myers G (2014). Efficient local alignment discovery amongst noisy long reads. Algorithms in bioinformatics WABI 2014 lecture notes in computer science.

[55] Langmead B, Salzberg SL (2012). Fast gapped-read alignment with Bowtie 2. Nat Methods.

[56] Kang DD, Froula J, Egan R, Wang Z (2015). MetaBAT, an efficient tool for accurately reconstructing single genomes from complex microbial communities. PeerJ.

[57] Walker BJ, Abeel T, Shea T, Priest M, Abouelliel A, Sakthikumar S, Cuomo CA, Zeng Q, Wortman J, Young SK (2014). Pilon: an integrated tool for comprehensive microbial variant detection and genome assembly improvement. PLoS One.

[58] Li D, Liu CM, Luo R, Sadakane K, Lam TW (2015). MEGAHIT: an ultra-fast single-node solution for large and complex metagenomics assembly via succinct de Bruijn graph. Bioinformatics.

[59] Hyatt D, Chen GL, Locascio PF, Land ML, Larimer FW, Hauser LJ (2010). Prodigal: prokaryotic gene recognition and translation initiation site identification. BMC Bioinformatics.

[60] Arumugam M, Raes J, Pelletier E, Le Paslier D, Yamada T, Mende DR, Fernandes GR, Tap J, Bruls T, Batto JM (2011). Enterotypes of the human gut microbiome. Nature.

[61] Merchant N, Lyons E, Goff S, Vaughn M, Ware D, Micklos D, Antin P (2016). The iPlant collaborative: cyberinfrastructure for enabling data to discovery for the life sciences. PLoS Biol.

[62] Paez-Espino D, Eloe-Fadrosh EA, Pavlopoulos GA, Thomas AD, Huntemann M, Mikhailova N, Rubin E, Ivanova NN, Kyrpides NC (2016). Uncovering Earth’s virome. Nature.

[63] Seemann T (2014). Prokka: rapid prokaryotic genome annotation. Bioinformatics.

[64] Kurtz S, Phillippy A, Delcher AL, Smoot M, Shumway M, Antonescu C, Salzberg SL (2004). Versatile and open software for comparing large genomes. Genome Biol.

[65] Mizuno CM, Valera-Rodriguez F, Kimes NE, Ghai R (2013). Expanidng the marine virosphere using metagenomics. PLoS Genet.

[66] Letunic I, Bork P (2016). Interactive tree of life (iTOL) v3: an online tool for the display and annotation of phylogenetic and other trees. Nucleic Acids Res.

[67] Thorvaldsdottir H, Robinson JT, Mesirov JP (2013). Integrative Genomics Viewer (IGV): high-performance genomics data visualization and exploration. Brief Bioinform.

[68] Zhu W, Lomsadze A, Borodovsky M (2010). Ab initio gene identification in metagenomic sequences. Nucleic Acids Res.

[69] Page AJ, Cummins CA, Hunt M, Wong VK, Reuter S, Holden MT, Fookes M, Falush D, Keane JA, Parkhill J (2015). Roary: rapid large-scale prokaryote pan genome analysis. Bioinformatics.

[70] Guy L, Kultima JR, Andersson SG (2010). genoPlotR: comparative gene and genome visualization in R. Bioinformatics.

[71] Le Chatelier E, Nielsen T, Qin J, Prifti E, Hildebrand F, Falony G, Almeida M, Arumugam M, Batto JM, Kennedy S (2013). Richness of human gut microbiome correlates with metabolic markers. Nature.

[72] Qin J, Li Y, Cai Z, Li S, Zhu J, Zhang F, Liang S, Zhang W, Guan Y, Shen D (2012). A metagenome-wide association study of gut microbiota in type 2 diabetes. Nature.

[73] Edgar RC (2007). PILER-CR: fast and accurate identification of CRISPR repeats. BMC Bioinformatics.

[74] Storey JD, Tibshirani R (2003). Statistical significance for genomewide studies. Proc Natl Acad Sci U S A.

[75] Finn RD, Clements J, Eddy SR (2011). HMMER web server: interactive sequence similarity searching. Nucleic Acids Res.

[76] Parks DH, Imelfort M, Skennerton CT, Hugenholtz P, Tyson GW (2015). CheckM: assessing the quality of microbial genomes recovered from isolates, single cells, and metagenomes. Genome Res.

[77] Pearson WR, Lipman DJ (1988). Improved tools for biological sequence comparison. Proc Natl Acad Sci U S A.

[78] Tamura K, Stecher G, Peterson D, Filipski A, Kumar S (2013). MEGA6: molecular evolutionary genetics analysis version 6.0. Mol Biol Evol.

[79] Katoh K, Standley DM (2013). MAFFT multiple sequence alignment software version 7: improvements in performance and usability. Mol Biol Evol.

