# Supplementary material for: Long-read metagenomic exploration of extrachromosomal mobile genetic elements in the human gut
Source: Microbiome. 2019 Aug 27;7:119. doi: 10.1186/s40168-019-0737-z (PMC6712665; doi:10.1186/s40168-019-0737-z)
Supplement: Supplementary file 1 — Figure S1. Genes in PacBio and short-read contigs. Figure S2. Optimization for identification of phage orthologous groups (POGs). Figure S3. Sequence alignments of two highly homologous but distinct plasmid CCs in three samples. Figure S4. Similarity search of 82 CCs against the public plasmid/phage database. Figure S5. Mapping of PacBio subreads and short reads to the five crAssphage CCs. Figure S6. Dot plot of terminal direct repeats in the five crAssphages. Figure S7. GC skews in the linear crAssphage genomes. Figure S8. Mapping of PacBio subreads to two phage CCs. Figure S9. Phylogenetic tree of 101 high-quality chromosome bins and 181 known genomes. Figure S10. Host prediction by methylation motif similarity between eMGEs and HQ chromosome bins in the PacBio JP dataset. Figure S11. Host-plasmid network. The predicted host-plasmid relationships were summarized and visualized as a network. Figure S12. Ratios of reads mapped to plasmids and crAssphages in 413 metagenomic data sets and proportions of crAssphage-positive individuals. Figure S13. Association analysis of the abundance of crAssphages with subjects’ age, BMI, and sex in the IGCJ dataset. Figure S14. Antibiotic resistance genes in plasmids in the IGCJ dataset. (PDF 6178 kb) [file 40168_2019_737_MOESM1_ESM.pdf]

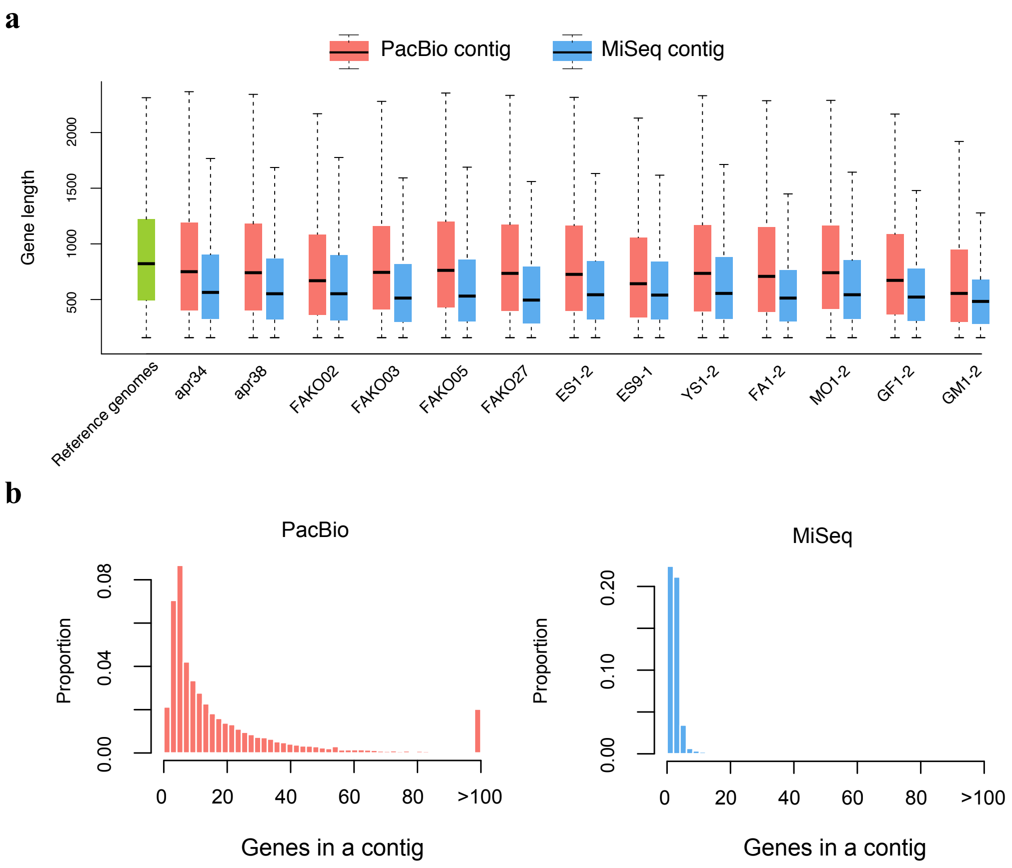


**Supplementary Fig. S1 | Genes in PacBio and short-read contigs.**

a, Comparison of length distributions of genes identified in PacBio and short-read (MiSeq) contigs and reference genomes containing complete genomes. The box plots show inter-quartile ranges (IQR) by boxes, medians by central lines, and the lowest and highest values within 1.5 times the IQR are shown by whiskers. For visualization, outliers are not shown in this figure. b, Histograms for the number of genes identified in the PacBio and MiSeq contigs.

a

| Identity | Alignment coverage | E-value | Sensitivity | Specificity |
|---|---|---|---|---|
| - | 20% | 1e-5 | 97% | 82% |
| - | 30% | 1e-5 | 97% | 84% |
| - | 40% | 1e-5 | 97% | 87% |
| - | 50% | 1e-5 | 97% | 90% |
| - | 60% | 1e-5 | 96% | 91% |
| - | 70% | 1e-5 | 96% | 92% |
| - | 80% | 1e-5 | 96% | 93% |
| - | 90% | 1e-5 | 95% | 95% |
| 20% | - | 1e-5 | 97% | 82% |
| 30% | - | 1e-5 | 97% | 83% |
| 40% | - | 1e-5 | 95% | 92% |
| 50% | - | 1e-5 | 90% | 95% |
| 60% | - | 1e-5 | 85% | 97% |
| 70% | - | 1e-5 | 80% | 98% |
| 80% | - | 1e-5 | 76% | 99% |
| 90% | - | 1e-5 | 73% | 99% |
| 20% | 20% | 1e-5 | 97% | 82% |
| 30% | 30% | 1e-5 | 97% | 85% |
| 40% | 40% | 1e-5 | 94% | 94% |
| 50% | 50% | 1e-5 | 89% | 97% |
| 60% | 60% | 1e-5 | 83% | 98% |
| 70% | 70% | 1e-5 | 79% | 99% |
| 80% | 80% | 1e-5 | 75% | 99% |
| 90% | 90% | 1e-5 | 72% | 99% |

b

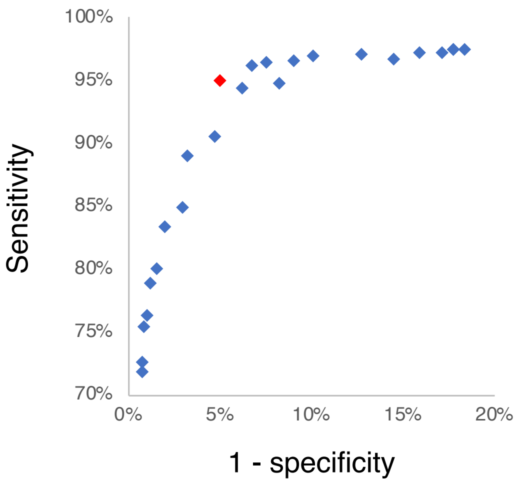


**Supplementary Fig. S2 | Optimization for identification of phage orthologous groups (POGs).**

a, Estimation of sensitivities (the number of phages from which POG(s) were detected / the number of phages) and specificities (the number of non-phages from which POG(s) were not detected / the number of non-phages) with various thresholds. Calculations were performed by aligning all predicted genes of the reference plasmid and phage sequences to phage orthologous groups (POGs). b, Relation between the sensitivity (y-axis) and the false positive ratio (1 – specificity). A red dot is the nearest to the perfect prediction at the upper left corner (100% sensitivity and 100% specificity) among the thresholds tested under the conditions of alignment coverage ≥90% without a threshold for identity.

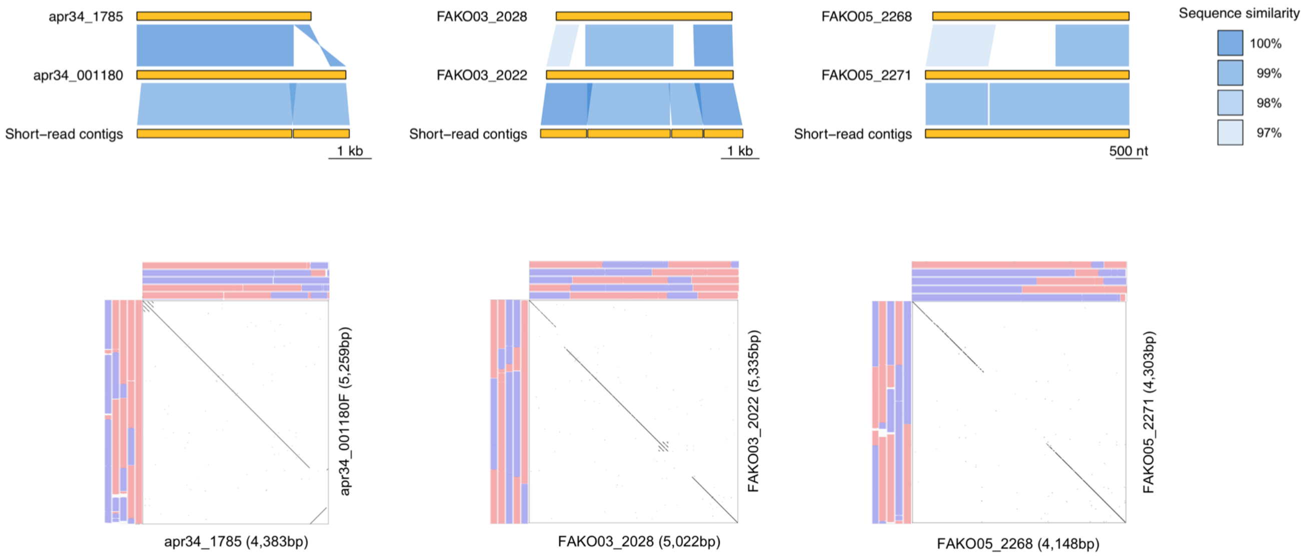


**Supplementary Fig. S3 | Sequence alignments of two highly homologous but distinct plasmid CCs in three samples.**

a, Alignment of three pairs of homologous plasmid CCs identified in three subjects. Orange bars represent two highly homologous plasmid CCs (upper two) generated from PacBio reads and the corresponding short-read contigs (bottom) in each sample. Multiple fragmented short-read contigs (left and middle) were aligned with the plasmid CCs, and a short-read contig (right) was aligned with one of either plasmid CCs. The homologous regions are connected with blue rectangles, of which shades indicate the degree of sequence similarity between them. b, Dot plots of two homologous plasmid CCs in three samples. PacBio subreads covering the forward and reverse strands of the entire CCs are shown by red and blue bars, respectively.

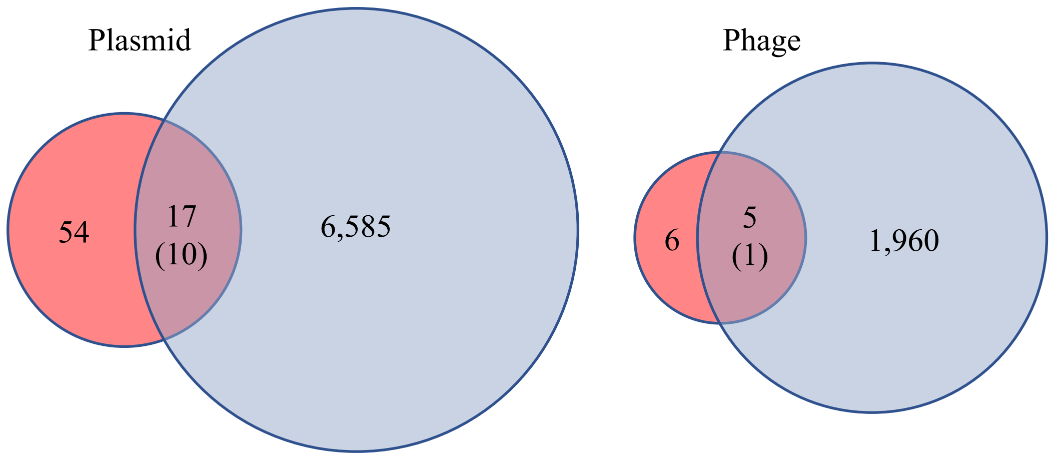


**Supplementary Fig. S4 | Similarity search of 82 CCs against the public plasmid/phage database.**

The Venn diagrams show 71 plasmid and 11 phage CCs (red) identified in this study and known plasmids and phages in GenBank (blue), respectively. The 17 plasmid CCs and five phage CCs were matched with 10 known plasmids and one phage (crAssphage) in GenBank, respectively.

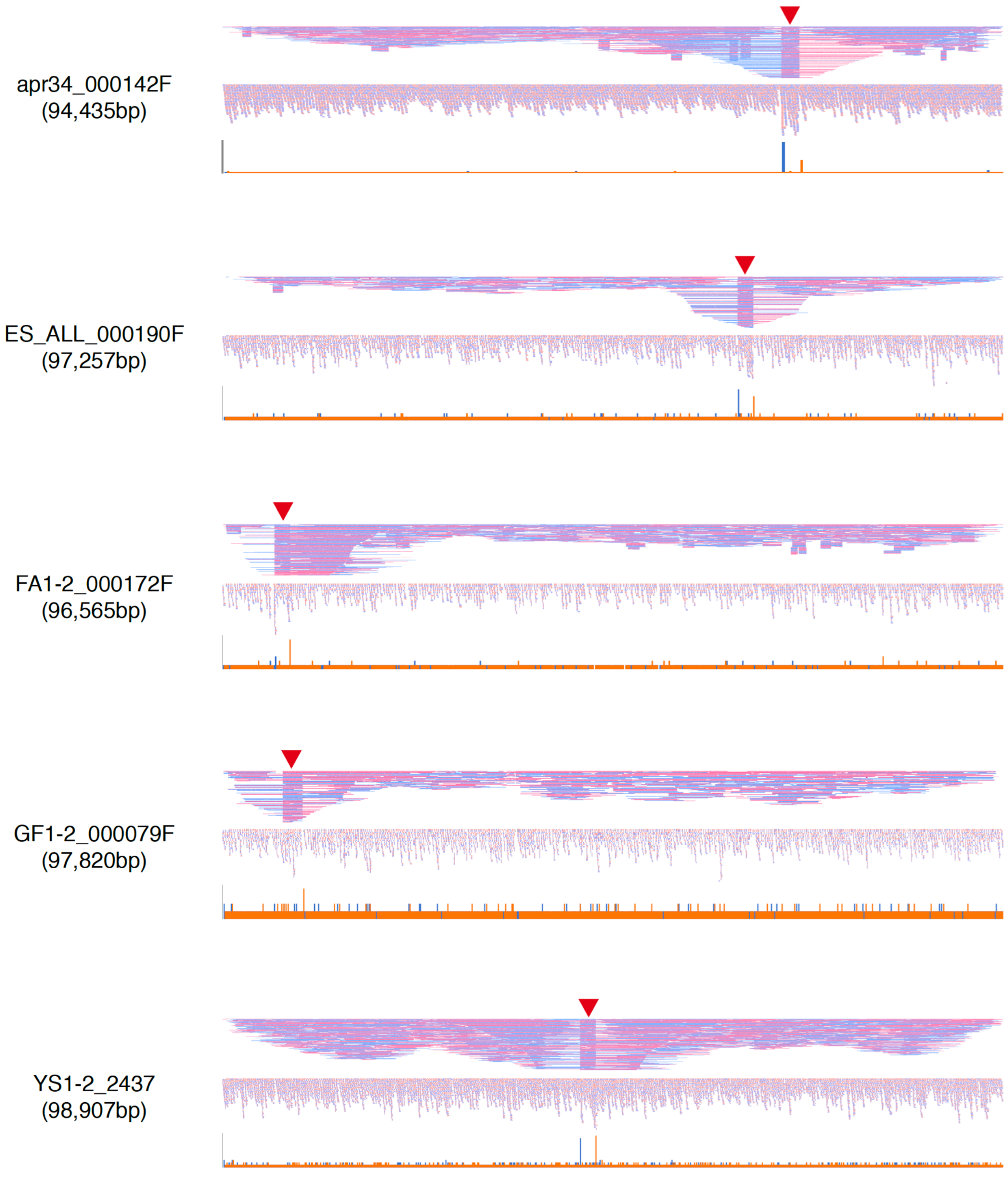


**Supplementary Fig. S5 | Mapping of PacBio subreads and short reads to the five crAssphage CCs.**

Alignments of PacBio long subreads and MiSeq short reads to the five crAssphage CCs are shown in the upper and middle diagrams, respectively. Red and blue horizontal lines represent forward and reverse reads mapped to the CCs, respectively. Red inverse triangles highlight the region of terminal direct repeats (TDRs) with approximately two times higher number of mapped reads than others in the CCs. Alignments with excessive PacBio subreads were eliminated from the diagrams. The bottom diagram shows the frequency of start sites (5´ position) of aligned short reads in the CCs. The orange and blue bars represent the numbers of forward and reverse reads, respectively.

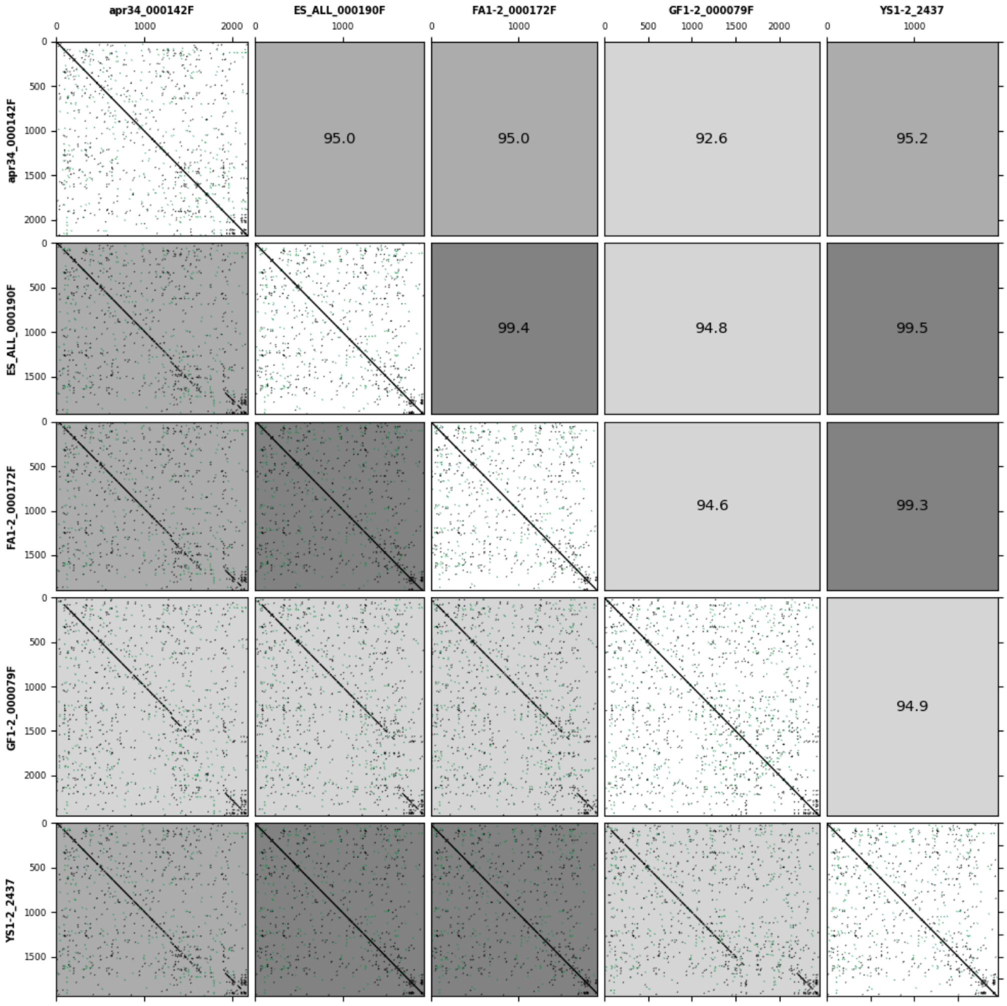


**Supplementary Fig. S6 | Dot plot of terminal direct repeats in the five crAssphages.**

Dot plots of all pairs of terminal direct repeats (TDRs) in the five crAssphage genomes are shown. The numbers in the matrix denote percentage identities between the two TDRs.

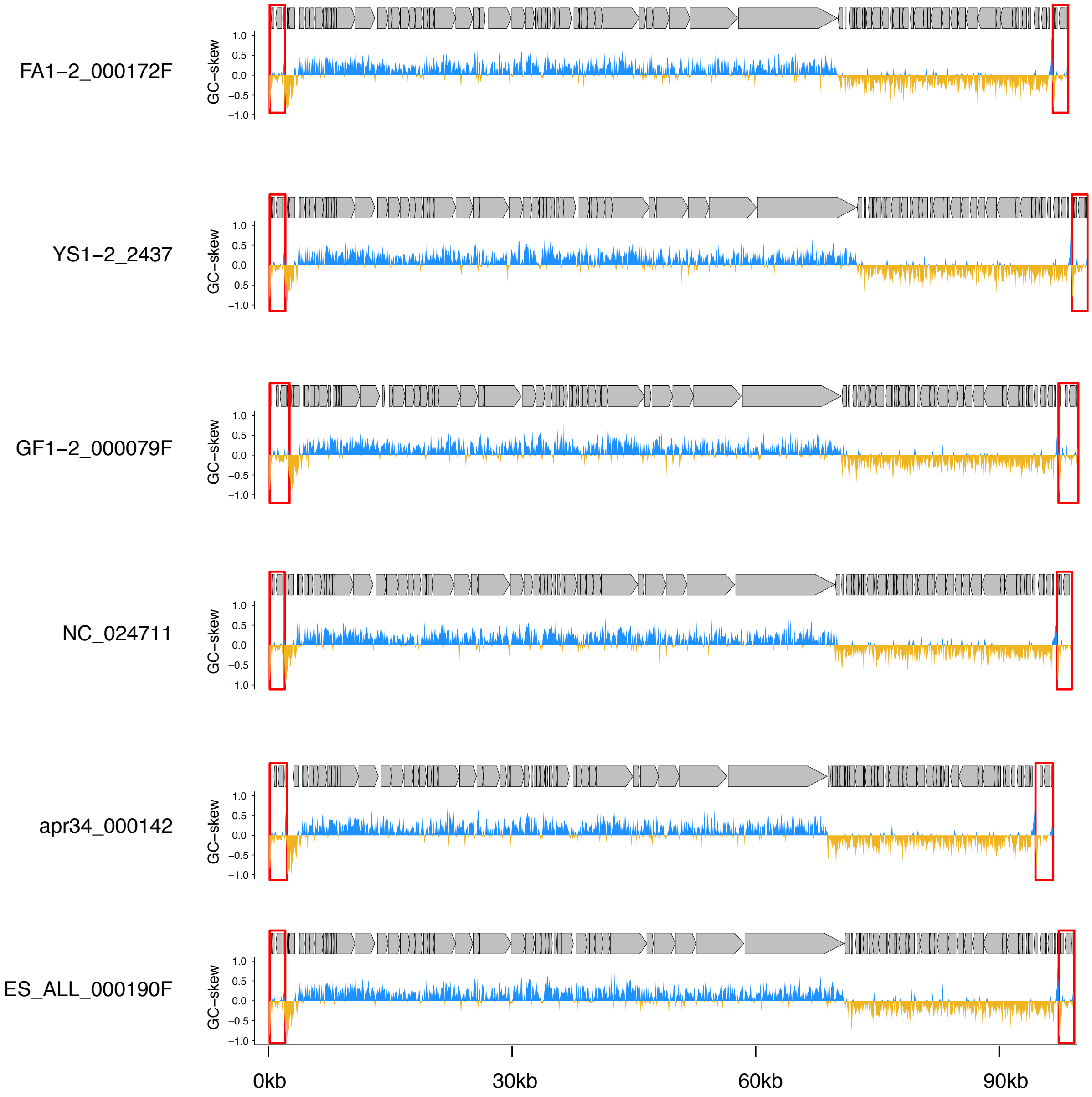


**Supplementary Fig. S7 | GC skews in the linear crAssphage genomes.**

Grey pentagons indicate putative genes in the crAssphage genomes. TDRs are indicated by red boxes. GC skews are shown in blue and orange.

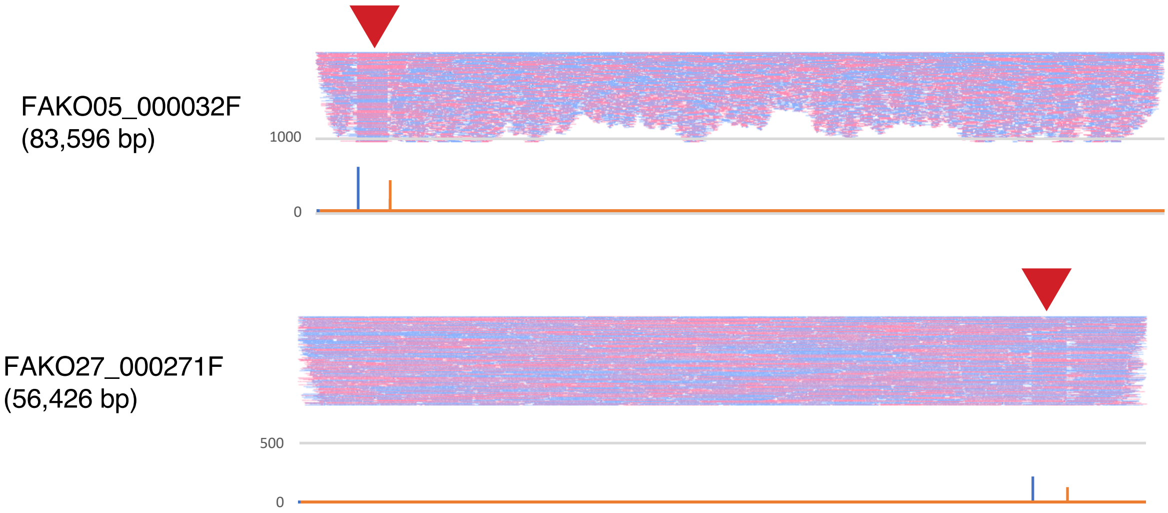


**Supplementary Fig. S8 | Mapping of PacBio subreads to two phage CCs.**

Alignments of PacBio subreads mapped to two phage CCs (FAKO05_000032F and FAKO27_0000271F) are shown as described in Supplementary Fig. S5. The data suggest that these two phage CCs have linear genomes.

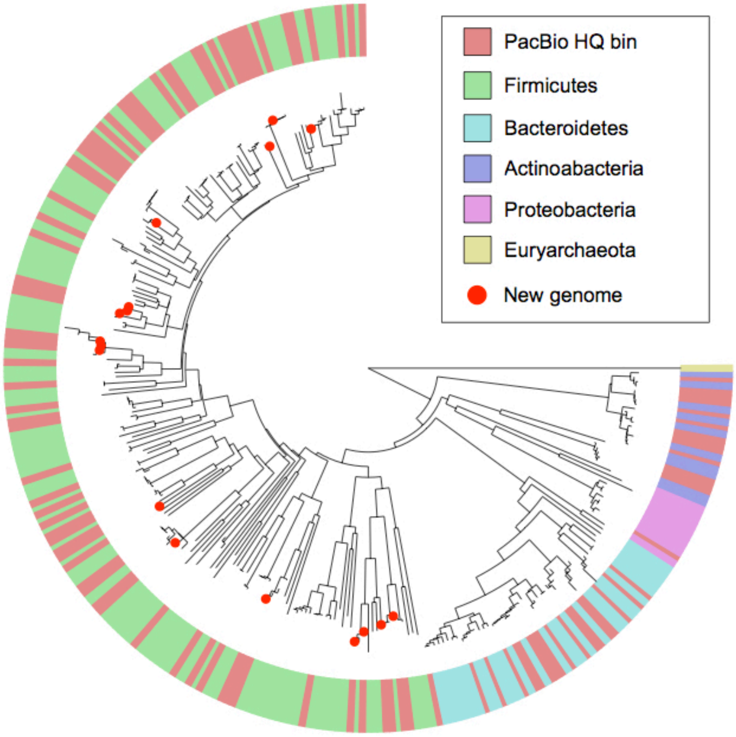


**Supplementary Fig. S9 | Phylogenetic tree of 101 high-quality chromosome bins and 181 known genomes.**

A neighbour-joining phylogenetic tree was constructed from 101 high-quality (HQ) genome bins and 181 known genomes of four phyla in GenBank with Euryarchaeota (Methanobrevibacter smithii) as an outgroup. Five phyla are shown in different colours (green for Firmicutes, purple for Actinobacteria, pink for Proteobacteria, blue for Bacteroidetes, and yellow for Euryarchaeota), and red for 101 HQ genome bins in the outer circle. Red circles on the tree edges indicate 17 novel genomes phylogenetically distinct from the known genomes.

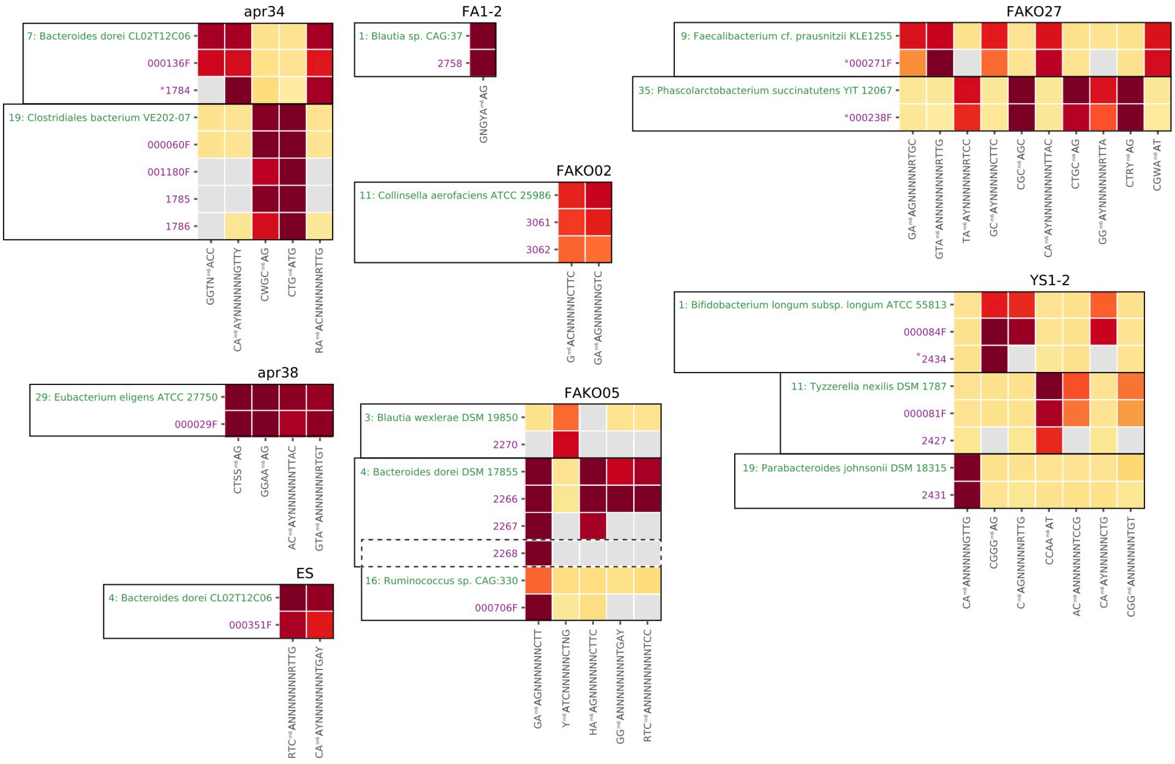


**Supplementary Fig. S10 | Host prediction by methylation motif similarity between eMGEs and HQ chromosome bins in the PacBio JP dataset.**

The eMGEs (purple) and host strains (green) having common methylation motifs (MMs) in eight subjects are shown with separate boxes. The eMGEs marked with asterisks indicate phages, and others are plasmids. The MMs with methylated adenines (m6A) are shown at the bottom of each box. Red shades indicate mean IPD ratio values higher than the threshold of 2.5, and yellow indicates mean IPD ratios less than the threshold. Grey denotes the absence of common MMs between host strains and eMGEs. The eMGE 2268 links with two different host strains are boxed by a dashed line.

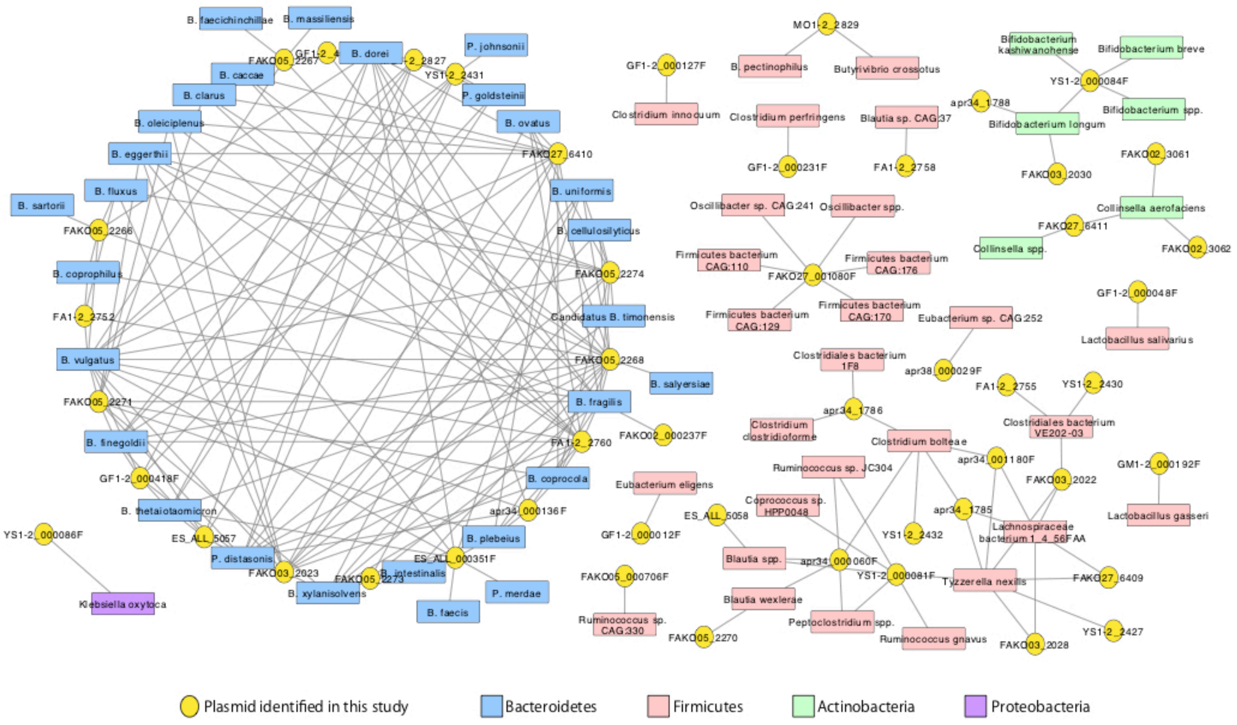


**Supplementary Fig. S11 | Host-plasmid network.**

The predicted host-plasmid relationships were summarized and visualized as a network. The circles and squares show plasmid CCs identified in this study and predicted hosts, respectively. The colours of the squares indicate host taxonomy at the phylum level (pink for Firmicutes, green for Actinobacteria, purple for Proteobacteria, blue for Bacteroidetes).

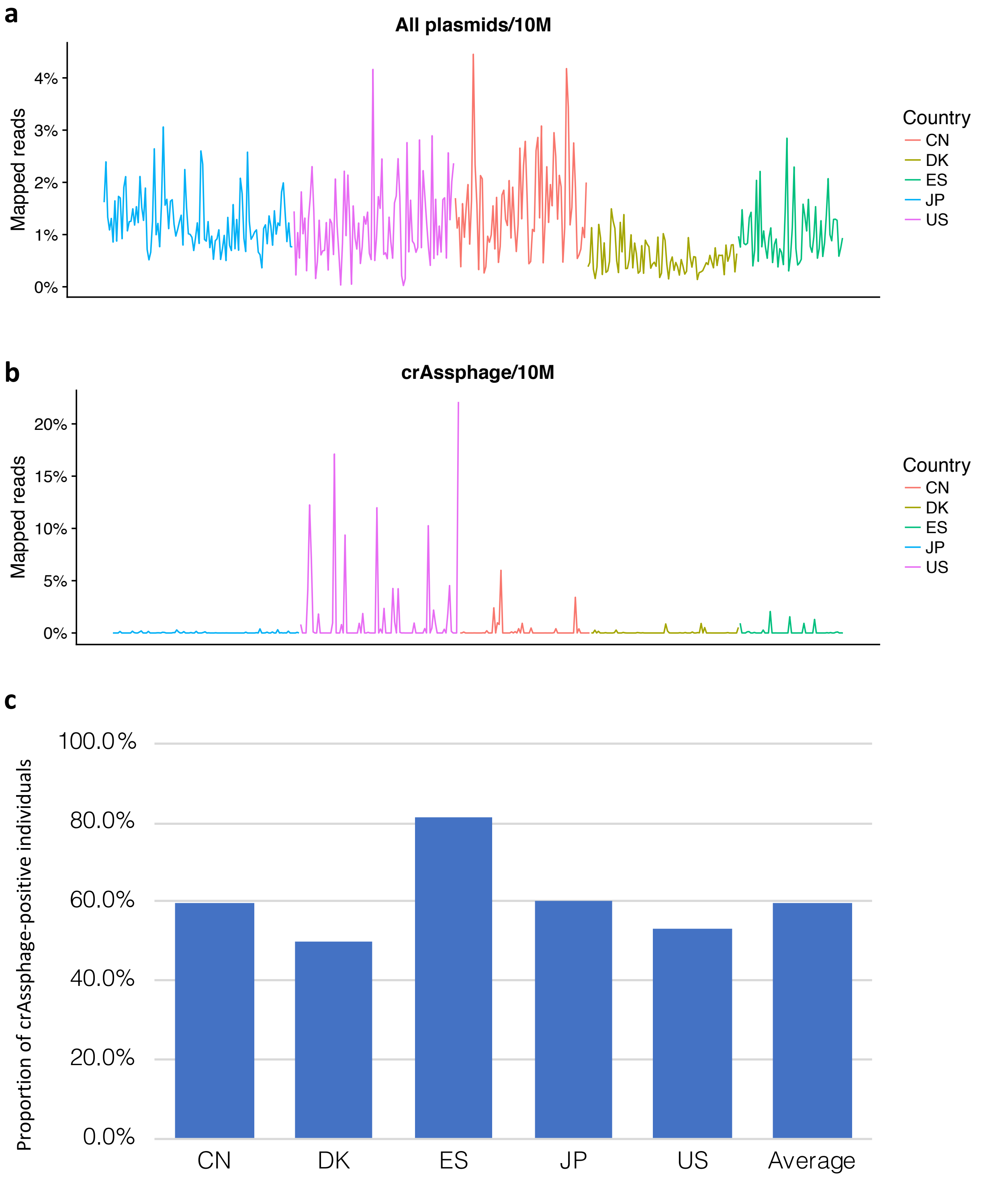


**Supplementary Fig. S12 | Ratios of reads mapped to plasmids and crAssphages in 413 metagenomic data sets and proportions of crAssphage-positive individuals.**

a, b, Metagenomic reads from 413 individuals (10 M reads per individual) in the IGCJ dataset are mapped to eMGE and crAssphage clusters. The x-axis shows 413 individuals from China (orange), Denmark (brown), Spain (green), Japan (light blue), and the US (pink). The y-axis shows the ratio of reads mapped to the clusters. c, Proportions of crAssphage-positive individuals (≥1 mapped read) in the five countries.

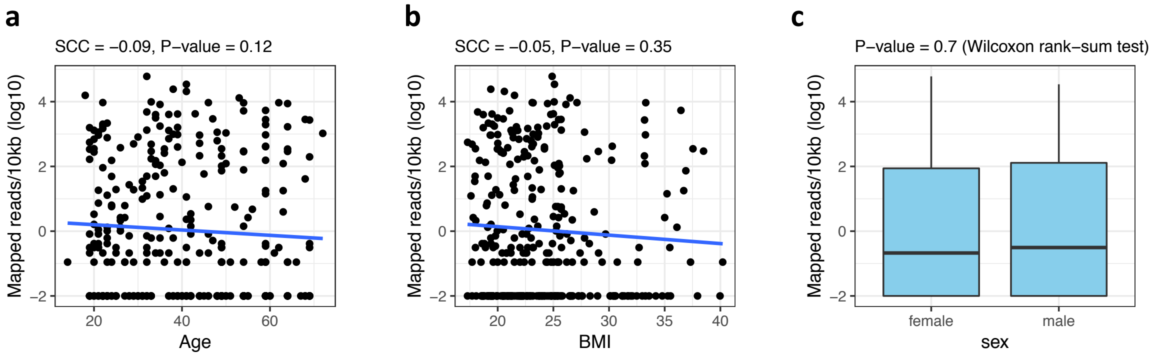


**Supplementary Fig. S13 | Association analysis of the abundance of crAssphages with subjects' age, BMI and sex in the IGCJ dataset.**

Spearman's correlation coefficients (SCCs) between crAssphage abundance and age (**a**) and BMI (**b**). Each circle represents each subject, and the blue line is the regression line. Comparison of crAssphage abundance between male and female participants (**c**). Pseudo-count (0.00001) was added to the abundance, and the values were log-transformed. The publicly available metadata (age, BMI, and sex) of 323 subjects in four countries (except the US subjects) were used for the analysis.

**a**

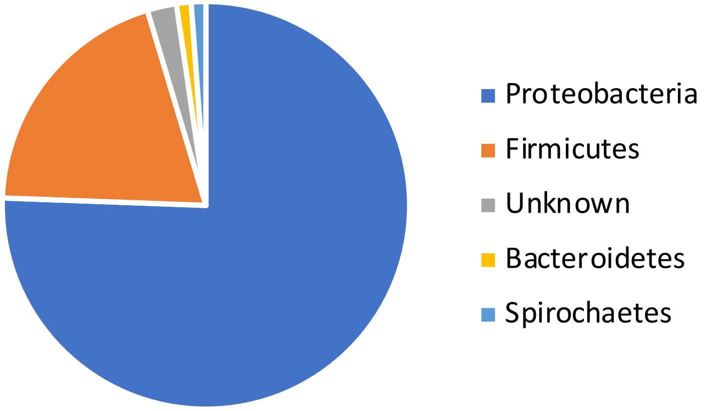


**b.**

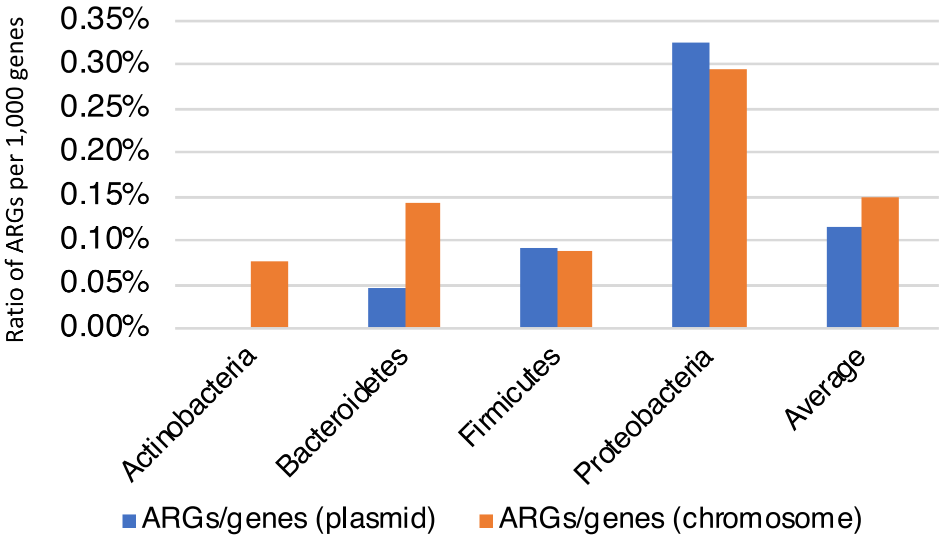


**Supplementary Fig. S14 | Antibiotic resistance genes in plasmids in the IGCJ dataset.**

a, Proportion of host phyla of plasmids containing antibiotic resistance genes (ARGs) based on the Resfams database. b, Ratio of ARGs per 1,000 genes in plasmids and chromosomes is shown.
